# Auxiliary ATP binding sites support DNA unwinding by RecBCD

**DOI:** 10.1038/s41467-022-29387-1

**Published:** 2022-04-04

**Authors:** Rani Zananiri, Sivasubramanyan Mangapuram Venkata, Vera Gaydar, Dan Yahalom, Omri Malik, Sergei Rudnizky, Oded Kleifeld, Ariel Kaplan, Arnon Henn

**Affiliations:** 1 Faculty of Biology, Technion - Israel Institute of Technology, Haifa, 3200003 Israel; 2 Russell Berrie Nanotechnology Institute, Technion - Israel Institute of Technology, Haifa, 3200003 Israel

**Keywords:** Enzyme mechanisms, Kinetics, Single-molecule biophysics, Optical tweezers, Mass spectrometry

## Abstract

The RecBCD helicase initiates double-stranded break repair in bacteria by processively unwinding DNA with a rate approaching ∼1,600 bp·s^−1^, but the mechanism enabling such a fast rate is unknown. Employing a wide range of methodologies — including equilibrium and time-resolved binding experiments, ensemble and single-molecule unwinding assays, and crosslinking followed by mass spectrometry — we reveal the existence of auxiliary binding sites in the RecC subunit, where ATP binds with lower affinity and distinct chemical interactions as compared to the known catalytic sites. The essentiality and functionality of these sites are demonstrated by their impact on the survival of *E.coli* after exposure to damage-inducing radiation. We propose a model by which RecBCD achieves its optimized unwinding rate, even when ATP is scarce, by using the auxiliary binding sites to increase the flux of ATP to its catalytic sites.

## Abstract (web summary)

RecBCD is a remarkably fast DNA helicase. Using a battery of biophysical methods, Zananiri et. al reveal additional, non-catalytic ATP binding sites that increase the ATP flux to the catalytic sites that allows fast unwinding when ATP is scarce.

## Introduction

Double strand breaks (DSBs) are the severest type of DNA damage in all kingdoms of life. Ubiquitous repair mechanisms for DSBs are found in every living organism, wherein helicases play an essential role. In prokaryotes, members of the RecBCD family initiate unwinding of the DNA at the DSB site in preparation for strand invasion, which is essential for the homologous recombination repair pathway^1,2^. RecBCD is a heterotrimer consisting of one copy each of the RecB and RecD DNA translocases and helicases of opposing unwinding polarity^3,4^. The RecC subunit “staples” the RecB and RecD subunits^5^, harbors the “pin” domain that has been proposed to split the duplex^5^, and recognizes the Chi sequence^6,7^. Previous studies have provided a wealth of knowledge on RecBCD’s biochemistry and structure^1,8–13^ and revealed important features of the unwinding process, including its initiation, kinetic step size, and inter-subunit regulation^12,14–18^.

RecBCD is a highly processive helicase exhibiting an exceptionally high unwinding rate of ∼ 1600 base pairs (bp) per second (s^−1^)^19^. Given that it consumes two ATP molecules per DNA bp unwound^20,21^, this amasses to a lower limit of 3200 hydrolyzed ATPs s^−1^ RecBCD^−1^, meaning that RecBCD is able to complete three ATPase cycles in less than a millisecond. Thus, while previous studies focused mainly on delineating the order of events that encompass the unwinding and translocation reaction, we emphasize that it is also essential to elucidate the specific properties allowing RecBCD to achieve its rapid catalytic cycle. In this work, we used a combination of biophysical assays to underpin the molecular mechanism for RecBCD’s superfast catalysis. Unexpectedly, equilibrium binding assays mapped previously unidentified nucleotide-binding sites in RecC, that are distinct in their affinity and kinetics of binding and in their sensitivity to ATP-analogs and salt. Using real-time, single-turnover, and single-molecule optical tweezers DNA unwinding assays, we found that the lower affinity sites contribute significantly to both the ATPase rate and the unwinding velocity, but only at ATP concentrations that are much higher than those required to fully saturate the canonical binding sites in both subunits. In addition, both binding and dissociation of ATP at these sites are required to achieve the maximum catalytic rate. A combination of nucleotide cross-linking followed by mass spectrometry and computational studies was used to map the auxiliary sites to specific locations in the RecC subunit, which were mutated to produce a variety of RecBC^mut^D proteins. Equilibrium nucleotide-binding and DNA unwinding assays with these proteins, and an in vivo survival assay with bacteria containing them, confirmed the identification of the auxiliary sites binding sites, and their functional role in the catalytic cycle of RecBCD. A model where ATP binding to the auxiliary binding sites serves to increase the flux of ATP to the catalytic sites fully recapitulates our binding and activity measurements.

## Results

### RecBCD possesses strong and weak nucleotide-binding sites

**Fig. 1:**
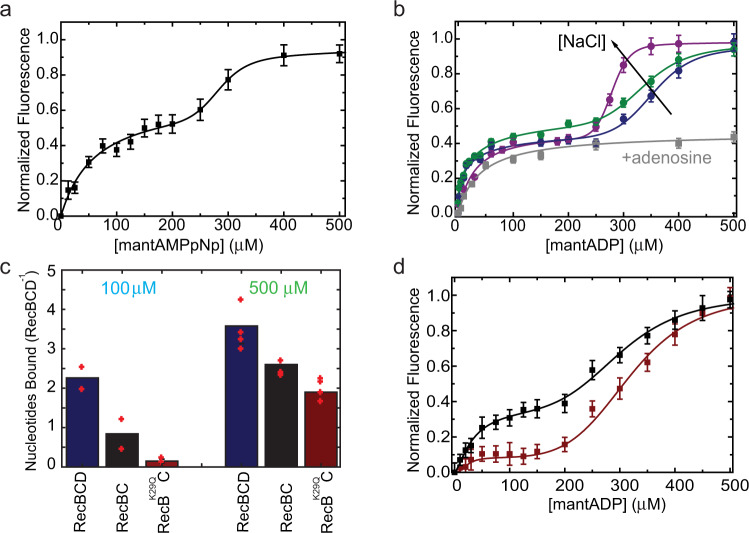
Equilibrium nucleotide binding to RecBCD. **a** Titration curves of mantAMPpNp to RecBCD exhibit a biphasic pattern reaching saturation at ∼500 μM nucleotide concentration. Lines show the best fit to Eq. 1 (Methods). Data are shown as mean ± s.e.m., *n* = 3. **b** Salt and adenosine dependence of mantADP binding to RecBCD. Blue, green, and purple correspond to 75, 200, and 300 mM NaCl, respectively. The gray line represents the binding curve in the presence of 75 mM NaCl and 2 mM adenosine and was multiplied by 0.4 to guide the eye. Data are shown as mean ± s.e.m., *n* = 3. Lines are best fits to Eq. 1 (Methods). **c** Number of ADP molecules bound to RecBCD (blue), RecBC (black), and RecB^K29Q^CD (red), measured using equilibrium dialysis, for low (∼100 µM, left) and high (∼500 µM, right) nucleotide concentrations. Data are shown as the mean of *n* = 2 and 4 independent measurements, for low and high concentrations, respectively. **d** Titration curves of mantADP to RecBC (black) and RecB^K29Q^C (red). Lines show the best fit to Eq. 1 (Methods). Data are shown as mean ± s.e.m., *n* = 3.

The ADP and ATP affinities of RecBCD were determined by Förster Resonance Energy Transfer (FRET) between the protein’s intrinsic tryptophans and fluorescent nucleotide analogs, mantADP, and mantAMPpNp, respectively. Remarkably, the pattern by which RecBCD binds to mant-nucleotides follows a biphasic behavior as a function of the nucleotide concentration (Fig. 1a, b). This biphasic behavior is not caused by impurities, as verified by performing mant-nucleotide-binding with proteins subjected to an additional MonoQ purification step (Supplementary Fig. 16). The competitive binding of unmodified ADP to a pre-equilibrated RecBCD·mantADP complex yielded a similar biphasic pattern (Supplementary Fig. 1), reinforcing the observed behavior. Since binding of a ligand to a macromolecule containing two binding sites cannot result in this type of biphasic pattern (Supplementary Note 1 and Supplementary Fig. 2), our results support the existence of two binding pathways: one hyperbolic with strong affinity, and one sigmoidal with weak affinity. The sigmoidal phase can only be observed if there are at least two cooperative sites that are distinct from the site/s of the first phase, so this accumulates to, at least, three nucleotide-binding sites within RecBCD. As a phenomenological characterization, we describe the binding isotherms as the sum of two Hill equations (Eq. 1, Methods). The first Hill curve describes stronger affinity, but weakly cooperative binding and is characterized by a macroscopic dissociation constant *K*_s_ and a Hill coefficient *n*_s._ The second Hill curve describes weaker affinity, but cooperative nucleotide-binding sites, with a dissociation constant *K*_w_ and Hill coefficient *n*_w_. The results of fitting such a model to the binding of mant-nucleotides are summarized in Supplementary Table 1.

### At least two non-catalytic binding sites are located at RecC or RecB

The equilibrium binding experiments reveal the existence of additional binding sites, but not their number. Moreover, they do not reveal the relative stoichiometry between sites corresponding to the different binding phases, since these sites may involve a different binding mode and hence a different FRET efficiency. Hence, to quantify the number of nucleotide-binding sites on RecBCD we employed equilibrium dialysis, a first principle, and model-free method. The experiments were performed at low nucleotide concentration (200 μM pre-equilibration; Supplementary Fig. 3a), where we aimed to saturate only the hyperbolic binding phase in our measured isotherms, and at high concentration (1 mM pre-equilibration; Supplementary Fig. 3b), at which we aimed to saturate both binding phases. In the low nucleotide concentration regime, RecBCD binds *n* = 2 ATP molecules/RecBCD (RecBCD·ADP: *n* = 2.26 ± 0.30; RecBCD·AMPpNp: *n* = 1.94 ± 0.15; mean ± s.e.m.; *N* = 2; Fig. 1c and Supplementary Fig. 3D). However, at high nucleotide concentration we found *n* = 3.5–4 ATP molecules/RecBCD for all the complexes measured (RecBCD·ADP: *n* = 3.58 ± 0.45; RecBCD·AMPpNp: *n* = 3.48 ± 0.70; RecBCD·ohDNA·ADP: *n* = 3.99 ± 0.50; mean ± s.e.m.; *N* = 4). We note that the high concentration experiments were performed at sub-stoichiometric concentrations (limited by [RecBCD]) and may not represent full saturation of all ligand binding sites. Hence, our results provide a lower limit on the number of binding sites and suggest that the strong binding phase accounts for two binding sites and the weak binding phase represents binding to at least two additional sites.

RecBC, a construct lacking the RecD subunit, can be well expressed and used to narrow down the location of potential additional sites. Figure 1d shows that RecBC binds to mant-nucleotides in a biphasic manner as well. Interestingly, the relative contribution of the strong binding phase decreased in this case, with only one catalytic site and about half the ATPase activity (Supplementary Fig. 3c) as compared with RecBCD (Supplementary Table 1). Consistently, equilibrium dialysis showed a reduction in one binding site at both low and high nucleotide concentrations (Fig. 1c). Remarkably, when we repeated these experiments with the catalytically deficient mutant RecB^K29Q^C, we found that the binding isotherms show a significant reduction in the strong binding phase (Fig. 1d), and the equilibrium dialysis shows practically no binding at low nucleotide concentration (Fig. 1c). Together, these results suggest that the strong binding phase corresponds to nucleotide binding to the two catalytic sites, and the weak binding phase to additional (at least two) sites, which are in the RecB or RecC subunits, or in both.

### ATP binds the strong and weak sites through distinct chemical interactions

To compare the biochemical properties of the additional nucleotide-binding sites to the canonical ones, we measured the binding isotherms as a function of NaCl concentration. We found that salt affects mainly the second phase of the binding curve, with an undistinguishable effect on the first (Fig. 1b and Supplementary Table 1). This suggests that binding to the weak sites is largely mediated by hydrophobic interactions, which are strengthened by salt^22^. We then examined the effect of adenosine nucleosides (nucleotides without the phosphate group) on mantADP binding to RecBCD. Excitingly, the presence of 2 mM adenosine specifically inhibited mantADP binding to the weak binding sites but showed a similar *K*_S_ as the one measured in the absence of adenosine (Fig. 1b). These results indicate that the chemical nature of the strong and weak binding sites is different, with ATP interacting with the weak binding sites mainly through the base and sugar moiety of the nucleotide. Furthermore, they suggest that adenosine and salt can be used as experimental tools to specifically modulate one set of binding sites in RecBCD.

### RecBCD’s individual subunits are active at similar ATP concentrations

**Fig. 2:**
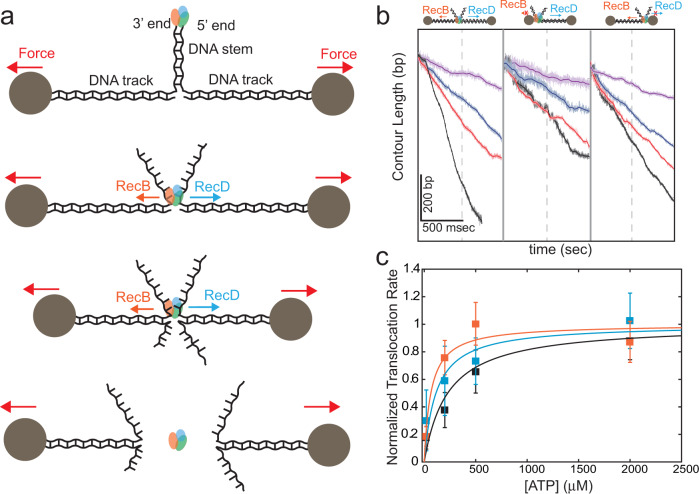
Single-molecule measurements of translocation by RecBCD and its individual subunits. **a** Schematic representation of the experimental optical tweezers setup. RecBCD binds to and translocates on a DNA stem connected to beads through DNA “tracks”. Upon reaching the fork, the helicase subunits translocate in different directions due to their opposing polarities, shortening the tether length. The force increases up to a point where RecBCD dissociates from the construct. **b** Representative traces of translocations on three constructs probing both translocases (left, symmetric tracks of 600 bp), RecD (middle, asymmetric tracks of ∼30 nt/∼4000 bp), and RecB (right, asymmetric tracks of ∼4000 bp/∼30 nt) at different ATP concentrations (black, 2 mM; red, 500 μM; blue 200 μM; purple, 50 μM). Light colors display the raw, 2.5 kHz data, and bold lines a moving average of 100 points. **c** Translocation rates versus [ATP] for both translocases (black), RecD (blue) and RecB (orange) in the force range 10–15 pN. Solid lines through the data points are fits to hyperbolic curves. Data are shown as mean ± s.e.m., normalized to the maximal rate according to the fit. The number of traces used in the analysis is summarized in Supplementary Table 2).

If RecB and RecD have significantly different affinities towards nucleotides, one would expect to find that, at an intermediate ATP concentration, one subunit will be fully active while the second will not. To study the activity of the individual subunits we exploited a recently developed single-molecule optical tweezers assay^18^. Figure 2a shows a schematic representation of the experiment, where a DNA construct, consisting of a stem with a blunt end attached to two dsDNA “tracks”, is tethered between two beads trapped in separate optical traps. Upon introduction of RecBCD in the presence of ATP, the enzyme binds to the blunt end and translocates until it reaches the fork. Then, due to the opposite polarities of RecB and RecD, each of the subunits translocates on an opposite track in an inter-subunit “tug-of-war”, as evidenced by an increase in the tether’s tension. As the force increases beyond a certain level (*F* *≈* 42–50 pN), RecBCD dissociates from the construct. By introducing asymmetry in the tracks’ length, such that one subunit reaches the bead in a considerably shorter time, we can directly measure the activity of individual subunits in the WT RecBCD in the context of the whole enzyme. Figure 2b shows representative traces in three experimental setups to probe the activities of both subunits (left), RecD (middle) and RecB (right) in the force range of 10–15 pN. Velocity curves calculated from all the translocation traces (Fig. 2c and Supplementary Table 2) indicate that the full enzyme, as well as its subunits, display an apparent hyperbolic dependence on ATP concentration. The measured *K*_1/2_’s are consistent with previously reported Michaelis-Menten fits for RecBCD^21,23^ and RecBC^11^. Remarkably, both of the *K*_1/2_’*s* values are significantly smaller than the nucleotide concentrations at which the second binding phase was observed (*K*_*w*_ *>* 280 µM, Supplementary Table 1). Since we measured similar affinities of RecBCD towards different nucleotides, our results indicate that both subunits’ catalytic sites are bound by ATP at concentrations that are lower than the weak binding phase, and further rule out the possibility of a biphasic curve arising from ATP binding to the two catalytic sites of each of the subunits of RecBCD. Corollary, they further support the identification of the higher affinity sites with RecBCD’s catalytic binding sites and the lower affinity sites with previously uncharacterized auxiliary binding sites for ATP.

Of note, the existence of a secondary translocase activity^11^ implies that the RecB subunit translocates in both the 3′ to 5′ and 5′ to 3′ direction. Hence, while the third construct of Fig. 2a probes the activity of RecB’s primary translocase, providing an upper bound for the *K*_D_ of RecB’s catalytic site, in the second construct two types of 5′ to 3′ activities are present: RecD’s translocase and the secondary translocase of RecB. Since both activities in RecB are derived from the same ATPase reaction^11^, these experiments set an upper bound on the *K*_D_’s of both RecB and RecD. Together, they lead to the same conclusions as stated above, without considering the secondary translocase activity of RecB.

### ATP binding to the strong and weak binding sites are kinetically separated

**Fig. 3:**
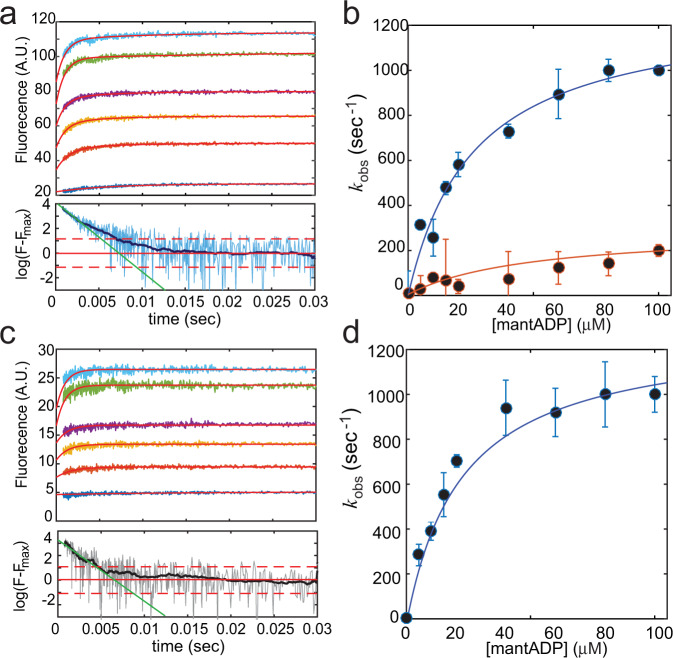
Transient kinetics of mantADP binding to RecBCD. **a** Top: Time courses of mantADP binding upon rapid mixing of RecBCD (2 µM, post-mixing) with mantADP (0–100 μM, lower to upper, respectively). The red lines through the data are the best global fit to a double exponential function (Methods). Bottom: Log-scale representation of the normalized intensity highlights the deviation from a single exponential. The light-blue line shows raw data of 60 μM and the dark-blue filtered data (Methods). Red horizontal lines indicate the mean (full line) and standard deviation (two dashed lines) of the noise. The green line is the best linear fit for the first 4 msec. In the time window from time t = 0 to the time when the signal equals the noise, up to a standard deviation, the data shows deviation from a single exponential decay. **b** Dependence of ${k}_{{{{{{{\mathrm{obs}}}}}}}}^{{{{{{{\mathrm{fast}}}}}}}}$ (Blue) and ${k}_{{{{{{{\mathrm{obs}}}}}}}}^{{{{{{{\mathrm{slow}}}}}}}}$ (Orange) on [mantADP], as obtained from fitting *n* = 7 independent measurements for each concentration. The error bars represent the standard error obtained from the fit. The solid line through the data points are the best hyperbolic fits. **c** Top: Time courses of mantADP binding upon rapid mixing of RecBCD (2 µM, post-mixing) with mantADP (0–100 μM, lower to upper, respectively) in the presence of 2 mM adenosine. The red lines through the data were the best global fit to a single exponential function (Methods). Bottom: Log-scale representation of the normalized intensity highlights a consistency with a single exponential. The gray line shows raw data of 60 μM and the black filtered data (Methods). Red horizontal lines indicate the mean (full line) and standard deviation (two dashed lines) of the noise. The green line is the best linear fit for the first 4 msec. **d** Dependence of *k*_obs_ on [mantADP] in the presence of adenosine, as obtained from fitting *n* = 7 independent measurements for each concentration. The error bars represent the standard error obtained from the fit. The solid line through the data points are the best hyperbolic fits.

Given the different chemical nature of the additional binding sites, we examined whether they also affect the kinetics of nucleotide binding to RecBCD. Unfortunately, the exceedingly rapid ATP turnover of RecBCD (one catalytic turnover <1 msec) implies that all biochemical transitions along the ATPase cycle occur at very fast rates, making the characterization of the binding kinetics very challenging. However, at 6°C, when the ATP turnover is significantly reduced (Supplementary Fig. 4) we were able to monitor the transient kinetics of mant-nucleotides binding to RecBCD (Fig. 3a–d, Supplementary Fig. 5, and Supplementary Table 3).

For all the concentrations measured, we observed two kinetic phases that can be well described by the sum of two exponential functions (Fig. 3a, b). The fast and slow phases (${k}_{{{{{{{\mathrm{obs}}}}}}}}^{{{{{{{\mathrm{fast}}}}}}}}$, ${k}_{{{{{{{\mathrm{obs}}}}}}}}^{{{{{{{\mathrm{slow}}}}}}}}$) exhibited a hyperbolic concentration dependence (Fig. 3b), indicating a minimal two-step binding mechanism where the initial binding is followed by an isomerization step that results in a high fluorescence state (Supplementary Note 2). Moreover, the saturation kinetics measured for the fast rate suggests that nucleotide binding to RecBCD takes place through parallel, independent pathways, rather than a single pathway of multiple, sequential binding events^24^.

To elucidate how the two phases observed in these experiments (“fast” and “slow”) correlate with the previously characterized binding sites (“strong” and “weak”), we measured the transient kinetics of binding in the presence of 2 mM adenosine. Remarkably, a single exponential phase was observed under these conditions (Fig. 2c), with a hyperbolic concentration dependence and similar values to the fast phase observed in the absence of adenosine (Fig. 2d). Hence, we conclude that the fast phase represents the binding of ATP to the strong, catalytic sites, while the slow phase represents binding to the weak sites.

### Modulating the auxiliary binding sites with adenosine and salt slows down RecBCD’s unwinding velocity

**Fig. 4:**
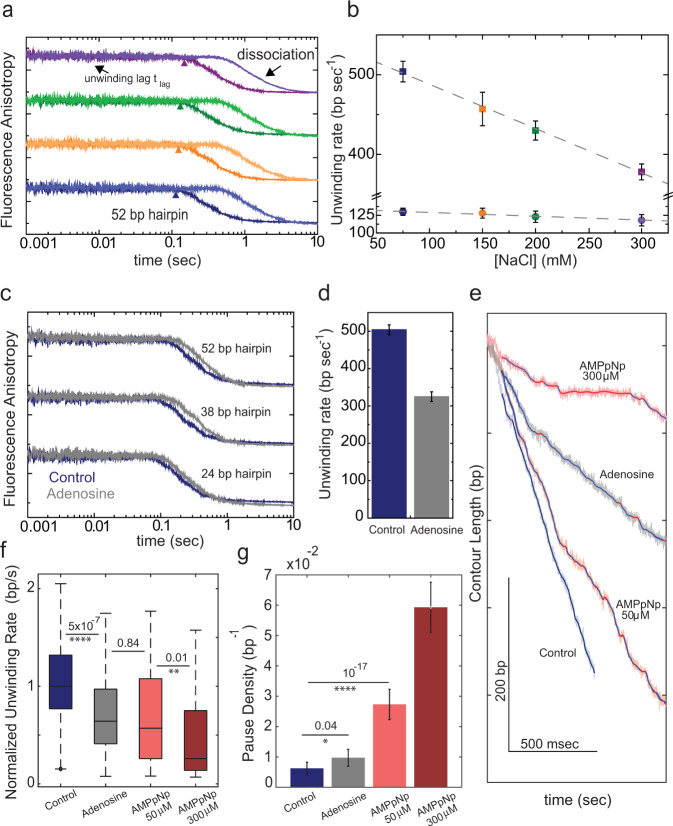
Rapid mixing, fluorescence anisotropy measurements of real-time unwinding by RecBCD. **a** FA time courses of unwinding reactions of RecBCD·hpDNA (52 bp; 250 nM, post-mixed) with ATP (350 μM, strong colors and 100 μM, light colors). Time courses were vertically shifted for clarity. Difference colors indicate time traces at 75 mM (blue), 150 mM (orange), 200 mM (green), and 300 mM (purple) NaCl concentrations. The curves are averages of ten traces. **b** Unwinding rates as a function of [NaCl], as calculated from the slopes in the lag-time vs. construct length curve (Supplementary Fig. 7C), for high (squares) and low (circles) [ATP]. Error bars indicate the standard error, as calculated from the fit. **c** FA time courses of unwinding reactions for a RecBCD·hpDNA complex (250 nM, post-mixed; top: 52 bp, middle: 38 bp, bottom: 24 bp) at [ATP] = 350 μM and [NaCl] = 75 mM, in the absence (blue) and the presence (gray) of 2 mM adenosine. Time courses were vertically shifted for clarity. The curves are averages of 10 (blue) and 15 (gray) traces. **d** Unwinding rate in the absence and the presence of 2 mM adenosine, calculated from the slope of lag-time vs. construct length curves. Error bars indicate the standard error, as calculated from the fit. **e** Representative traces of single-molecule unwinding/translocation experiments in the presence of adenosine (gray) and AMPpNp (light red, 50 μM and dark red, 300 μM), at 2 mM ATP. Also shown is a control (blue) in the absence of both adenosine and AMPpNp. Detected pauses are marked in red for all traces. **f** RecBCD’s normalized translocation rates in the absence and presence of adenosine or AMPpNp. Box-and-whiskers plot (median, IQR and min-max). *n* = 48, 127, 52, and 85 segments lasting 250 msec each, from 22, 46, 20, and 14 traces, for control, adenosine, 50 μM AMPpNp, and 300 μM AMPpNp, respectively. The numbers indicate *p*-values calculated with a two sample Student’s *t*-test, two-sided. ****p < 0.0001, **p < 0.01. Color coding as in **a**. **g** Pause density in the absence and the presence of adenosine and AMPpNp, calculated by dividing the total number of pauses in all traces (*tp*) by the total number of translocated base pairs (*tc*), using 22, 46, 20, and 14 different traces for control, adenosine, 50 μM AMPpNp and 300 μM AMPpNp, respectively. The error bars show 95% Poisson confidence intervals, i.e., ±1.96∙√(*tp*⁄*tc*). The numbers indicate *p*-values calculated with a two-sided Chi-squared test, ****p < 0.0001, *p < 0.05.

To characterize the functional role of the weak sites on the unwinding activity of RecBCD, we performed two different but similar real-time, single-turnover assays, based on fluorescence anisotropy (FA, Supplementary Fig. 6a) and FRET (Supplementary Fig. 6b). In both cases, preincubated RecBCD·DNA is rapidly mixed with ATP and ssDNA traps for the unwound DNA and the dissociated RecBCD. Time courses of the unwinding reactions exhibit a lag phase before the signal (FA or FRET) decays (Fig. 4a), which corresponds to the unwinding of the DNA and whose duration is proportional to the DNA length (Fig. 4a and Supplementary Fig. 7a, b). The slope of the lag-time vs. substrate length yields the unwinding rate (Supplementary Fig. 7c).

We followed the unwinding reaction in two ATP concentration regimes based on the equilibrium binding curves (100 and 350 µM), and with varying NaCl concentrations to differentially affect the different sites (Fig. 4a and Supplementary Fig. 7a, b). The intercept of *t*_lag*,*uw_ with the construct length axis remained constant regardless of the concentration of NaCl and ATP (Supplementary Fig. 7C), indicating that the complex formation time is very short, and was not significantly affected in the range of NaCl concentrations studied. At 75 mM salt, the unwinding rates measured for both 100 and 350 µM ATP are consistent to the ones previously reported measuring RecBCD’s translocation and unwinding in bulk^11,12^ and single-molecule assays^19,23^. The results show that at 350 µM ATP, when the weak binding sites are approximately half occupied and hence are sensitive to salt, the unwinding rate decreases as the concentration of NaCl is increased (Fig. 4b and Supplementary Table 4). However, at 100 µM ATP, when the weak binding sites are mostly unoccupied, salt did not affect RecBCD’s unwinding rate. These results support that binding to the weak sites is functionally important for RecBCD to attain a rapid unwinding rate at relatively high ATP concentration.

Given the facilitating effect of NaCl on nucleotide binding to the auxiliary sites (Fig. 1b), and the concurrent decrease in unwinding velocity (Fig. 4b), one may postulate that ATP binding to the weaker sites may play a negative allosteric function. Therefore, preventing binding to these sites by adenosine should increase the unwinding rate. However, when we compared unwinding velocities at 350 µM ATP in the presence and absence of 2 mM adenosine (Fig. 4c), we found that adenosine slowed down the unwinding velocities significantly (Fig. 4d), suggesting a slower mode for unwinding when the weaker binding sites are blocked, and implying a role for these sites, beyond a simple allosteric effect.

To examine the underlying mechanism by which adenosine slows down the velocity of RecBCD, we tested whether this is due to competition with ATP for the catalytic sites. We used our single-molecule assay to compare the effect of adenosine with that of AMPpNp. Figure 4e shows typical translocation traces at 2 mM ATP in the absence and the presence of adenosine (2 mM) or AMPpNp (50 μM and 300 μM). Remarkably, while AMPpNp induces ubiquitous pauses in the traces, an indication of a nonhydrolyzable analog bound at the catalytic site, adenosine slows down RecBCD without introducing measurable pauses. In particular, whereas the average velocity of translocation is comparable for 2 mM adenosine and 50 μM AMPpNp (Fig. 4f), and slower as compared to the ATP-only case, the slowing down is due to different mechanisms: the presence of AMPpNp results in an increase in the pause density, while for adenosine the slowing down occurs without significant pauses as compared to the ATP-only case (Fig. 4g), suggesting that the pause-free, instantaneous rate is decreased by preventing binding to the weak sites.

### Mutations in RecC hamper binding to the auxiliary sites and reduce the efficiency of DSB repair in vivo

**Fig. 5:**
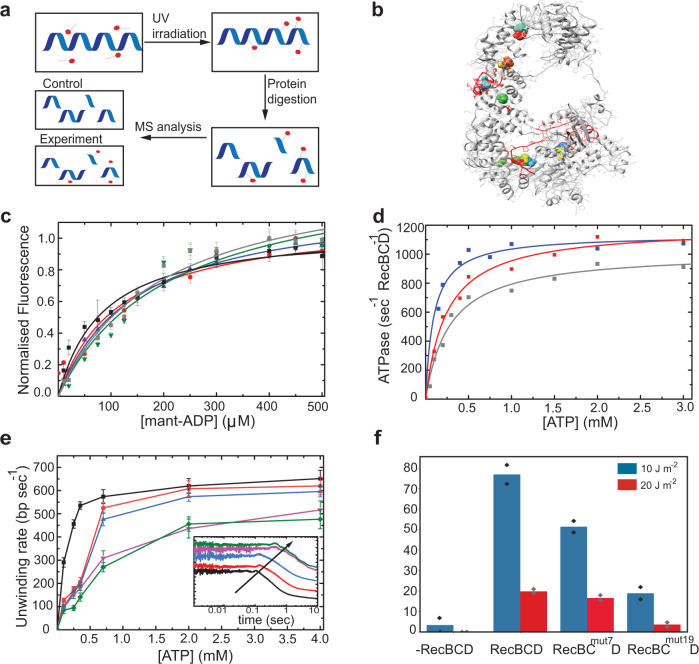
Identification of the auxiliary sites and characterization of RecBC^mut^Ds. **a** Cross-linking of RecBCD with 8-azido-modified ADP and ATP, followed by MS analysis (CL-MS) to identify the auxiliary ATP binding sites in the RecC subunit. **b** Structure of the RecC subunit from RecBC^mut19^D. The peptides from the CL-MS analysis and the mutations introduced were marked on the X-ray crystal structure of the RecC subunit (PDB-1W36) using Pymol. **c** Titration curves of mantADP to RecBC^mut4^D (Black), RecBC^mut7^D (Red), RecBC^mut13^D (Blue), RecBC^mut19^D (Green), and RecBC^mut19^D in the presence of 2 mM adenosine (Gray) exhibit a monophasic pattern. The binding between fluorescent nucleotide analog mantADP and RecBC^mut^D is measured as a Förster resonance energy transfer (FRET) signal between the intrinsic tryptophans (l_ext_ = 280 nm) and the mant-nucleotides (l_em_ = 436 nm). Data are shown as mean ± s.e.m., *n* = 3 independent experiments. **d** ATP concentration-dependence of RecBCD steady-state ATP turnover velocity (ATPase activity) of RecBCD (Blue), RecBCD^mut7^D (Red), and RecBC^mut19^D (Gray). The solid lines through the data points are the best fits to the quadratic form of the Briggs–Haldane equation (Eq. 2). **e** Unwinding rates of RecBCD (Black), RecBC^mut4^D (Red), RecBC^mut7^D (Blue), RecBC^mut13^D (Purple), and RecBC^mut19^D (Green) as a function of [ATP], calculated from the slope of lag-time vs. construct length curves, which were obtained by averaging *n* = 10 experimental traces. Error bars indicate the standard error, as calculated from the fit. The graph in the inset shows representative unwinding time courses of RecBCD and RecBC^mut^Ds (color-coded as in **c**). The arrow points to the increasing t_lag_ of the mutants. **f** Survival percentage of *E. coli* expressing WT, mutated, or lacking RecBCD (-RecBCD) following UV irradiation dosages of 10 J/m^2^ (Blue) and 20 J/m^2^ (Red). *n* = 2 independent experiments.

To identify the location of the auxiliary ATP binding sites, we performed biochemical cross-linking of RecBCD with 8-azido-modified ADP and ATP^25^, and analyzed the crosslinked peptide-nucleotides adducts by mass spectrometry (CL-MS, Fig. 5a) to map amino acids with a shifted molecular mass (Supplementary Table 5). Integrating also information from a computational search using FTMap (Supplementary Fig. 8), we identified regions in RecC with nucleotide-binding reactivity (Fig. 5b). Next, we designed mutations at these regions aimed at reducing binding of nucleotides by selecting amino acids with relatively less volume and in some cases also by changing the charge or hydrophobicity relativo to those present in WT RecBCD. Overall, we expressed and purified four mutant proteins (RecBC^mut^Ds), with different combinations of 19 point mutations in the RecC subunit (Supplementary Table 6). Remarkably, the pattern by which RecBC^mut^Ds bind to mant-nucleotides follows a hyperbolic (monophasic) binding isotherm as a function of the nucleotide concentration (Fig. 5c), and the low-affinity phase observed in the curve of RecBCD is no longer observed in the mutants’ binding isotherms. Moreover, the binding isotherms are insensitive to the addition of adenosine, indicating that the mutations in the identified auxiliary binding sites in the RecC subunit have a deleterious effect on the weak binding of ATP. The nucleotide affinity of RecBC^mut4^D and RecBC^mut7^D is lower as compared to the strong phase of WT RecBCD (Supplementary Table 9), which indicates that mutating the auxiliary binding sites in the RecC subunit has a weakening effect on the catalytic ATP binding sites residing in RecB and RecD. This may suggest some degree of allosteric interactions between the mutated auxiliary binding sites and the catalytic sites. Notably, constructs with a higher number of mutations, 13 and 19, displayed lower ATPase (Fig. 5d and Supplementary Table 7) and unwinding rates at saturating ATP concentration than the WT RecBCD (Fig. 5e and Supplementary Table 8), an indication that the mutations likely have a longer range effect on RecBCD inter-subunit interactions^26^, and thus a deleterious effect on its catalytic function. However, RecBC^mut^Ds with 4 and 7 mutations exhibited lower unwinding rates only up to ∼350 µM ATP, and then saturate at similar values as the WT (Fig. 5e). This suggests that these mutations perturb binding of ATP without affecting the subsequent steps in the hydrolysis reaction, and are therefore good probes for the role of the auxiliary sites. Interestingly, the mutated residues in these constructs, in particular RecBC^mut7^D, have a high degree of evolutionary conservation (Supplementary Fig. 9).

To further test if the auxiliary binding sites influence *E.coli’s* ability to cope with DSBs, we examined cells harboring RecBC^mut7^B in a survival assay. We utilized UV-C (254 nm) as an exogenic DSB-inducing agent^27,28^ and measured the number of colony-forming units (CFUs) in irradiated bacterial cultures relative to non-irradiated ones (Supplementary Fig. 10). RecBC^mut7^D displayed a significant and strong decrease in the survival probability (Fig. 5f), demonstrating that the efficiency of DNA damage repair in *E.coli* is dependent on the functionality of the auxiliary binding sites.

### A kinetic scheme that incorporates the transfer of ATP to the catalytic sites recapitulates the data

**Fig. 6:**
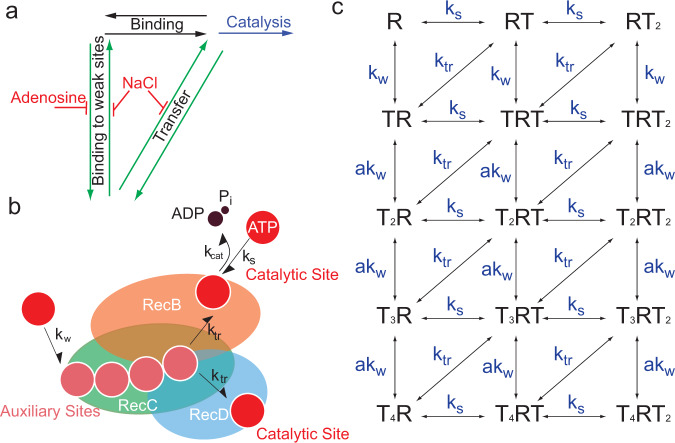
Transfer of ATP from RecBCD’s auxiliary sites to the catalytic ones supports rapid catalysis. **a**. Binding of ATP to RecBCD’s catalytic sites can be achieved through two parallel pathways: directly from the solution (black), or through cooperative binding to the auxiliary sites followed by a transfer step (green). **b**. RecBCD utilizes the auxiliary binding sites as an ATP buffer, facilitating binding to the catalytic sites. The RecBCD enzyme is represented by orange, green, and blue ovals for RecB, RecC, and RecD, respectively. **c**. Schematic representation of the chemical intermediates in our model. R represents RecBCD, T represents an ATP molecule. *T*_*n*_*RT*_*m*_, represents *n* ATP molecules bound to the non-catalytic (auxiliary) sites and *m* ATP molecules bound to catalytic sites. Binding to RecBCD catalytic sites is uncooperative with rate *k*_s_, binding to the non-catalytic sites, *k*_w_, is cooperative (binding to the second, third or fourth non-catalytic sites is faster by the factor *a*). Transfer from the non-catalytic sites to the catalytic sites occurs at a rate *k*_tr_. The reverse rates corresponding to each step are not shown, for clarity.

Our data indicate that blocking ATP binding to the weaker sites by adenosine lowers the unwinding velocity of RecBCD, therefore suggesting that binding to these sites plays a role in the catalytic cycle. In addition, since salt reduces the unwinding rate at high ATP concentration, it seems that the ability to dissociate from these sites is important as well, arguing against an allosteric mechanism of catalysis regulation by binding to the weak sites. Hence, we propose a model where ATP binds to the weak sites and transfers, perhaps by diffusion on the surface of the protein, to the catalytic site (Fig. 6a, b). The premises for such model are: (1) The catalytic sites are non-cooperative and identical, (2) There are additional, weak sites that are cooperative (for simplicity, we assume four such sites; Fig. 6c), (3) For simplicity, we assume no cooperativity/allostericity between the weak and the strong binding sites, (4) Unwinding can occur from any state that includes ATP in at least one of the catalytic sites, and (5) There is a transfer of ATP from the non-catalytic to the catalytic site sites.

Based on this scheme, we globally fitted (Methods, Supplementary Fig. 11 and Supplementary Table 10) our measurements (equilibrium and kinetics of nucleotide-binding and unwinding rates) with the complete set of kinetic parameters present in our model. Our global fitting analysis recapitulates all the data and reveals as expected that RecBCD’s additional binding sites are characterized by slow binding and dissociation rates, weak affinity binding to ATP, and sensitivity to adenosine and salt (Supplementary Table 10 and Supplementary Fig. 12). The analysis confirms that both binding and release from these sites are essential for the fast binding of ATP to the catalytic sites (Supplementary Fig. 13). In line with the hyperbolic kinetics measured for unwinding by RecBCD as a function of ATP concentration, here and previously^21,23^, the predicted unwinding rate as a function of ATP concentration shows a hyperbolic-like dependence, with and without adenosine, and not a biphasic behavior. Moreover, the model predicts (Supplementary Fig. 14) the observed reduction in *K*_M_ upon perturbation of the auxiliary sites (Fig. 5d).

## Discussion

RecBCD initiates the DSB response in *E. coli* by unwinding DNA at a remarkably fast rate. In our work, we aimed to elucidate what are the specific characteristics that allow RecBCD to catalyze the unwinding reaction at such high velocities. We revealed that in addition to the known catalytic sites, RecBCD harbors auxiliary nucleotide-binding sites of functional importance. Equilibrium binding titration curves exhibited a biphasic behavior that cannot be accounted by two catalytic sites only, and equilibrium dialysis experiments showed that RecBCD binds at least four nucleotides. Binding experiments in the presence of NaCl and adenosine allowed us to show that the chemical nature of the strong and weak binding sites is different. Transient kinetic experiments showed that binding of ATP to RecBCD catalytic and auxiliary sites occurs independently, and with different kinetic rates. Single-molecule experiments probing the translocation activity of the RecB and RecD subunits in the native context of a WT RecBCD complex showed that both subunits are active at similar ATP concentrations, allowing us to conclude that the strong sites correspond to the known catalytic ones, while the weak ones are previously uncharacterized binding sites for ATP. We then used cross-linking of RecBCD with 8-Azido-ATP to identify the location of putative binding sites and targeted such sites in RecC by different combinations of point mutations aimed at weakening binding. Two of the constructs probed, with 4 and 7 mutations, respectively, exhibited a similar ATPase and unwinding rate at saturating ATP concentration as the WT complex, but a lower effective binding rate and, remarkably, a monophasic mant-nucleotide-binding isotherm, supporting the identification of the mutated regions as auxiliary binding sites. *E. coli* cells containing mutated proteins were also deficient in DSB repair in vivo.

Several studies have shown, biochemically and/or structurally, potential nucleotide-binding sites, beyond the canonical Walker A-B catalytic sites, in helicases as well as in one myosin isoform^29–33^. However, no catalytic function was demonstrated for these sites. The most detailed study in this respect, PriA helicase, shows two unique sites, with high and low affinities^30^, which modulate the specificity for DNA structure. Secondary nucleotide sites have also been observed in the eIFA- eIF4G complex^31^ and Brr2 RNA helicase^33^. Hence, the growing evidence for the existence of secondary non-canonical ATP binding sites in other molecular motors may indicate a universal mechanism. However, the molecular mechanism by which they impact mechanochemical transduction during unwinding is yet to be revealed.

Our results support a mechanism for the rapid turnover of RecBCD at sub-optimal conditions, in which the newly characterized sites serve as a “buffer” of ATP molecules. ATP molecules bound at the auxiliary sites can quickly transfer to the catalytic sites upon release of the hydrolysis products resulting from the previous cycle. Such a mechanism effectively enhances the flux of ATP to the catalytic sites under a specific kinetic premise: The overall flux of ATP molecules from the auxiliary sites to the catalytic ones, which includes ATP binding to the auxiliary sites and its subsequent transfer to the catalytic sites, must be larger than the rate of binding directly from the solution. The global fitting results indicate that the transfer rate is indeed very fast (∼4 × 10^5^ sec^−1^). However, the rate of binding to the auxiliary sites is lower than binding directly to the catalytic ones, as evidenced also from the transient kinetic experiments. This apparent contradiction is settled by considering the auxiliary sites as highly cooperative. As a result, the rate constant for ‘replenishing’ the buffer is given by *ak*_w_ which, since *a ∼*18, is larger than *k*_s_. Notably, the auxiliary sites may have a different degree of orientational selectivity than the catalytic sites, thus may play a role in reducing the rotational diffusion component of the binding time. Therefore, it is possible that the auxiliary sites serve a dual role, buffering and steering ATP to the catalytic sites. And, while a purely allosteric effect of the auxiliary sites on the catalytic ones cannot explain our data, and the transfer of ATP molecules between them is at the core of our results, we also cannot rule out that such an effect plays a certain role. The newly discovered role of ATP as a biological hydrotrope in solubilizing and stabilizing proteins by binding in a non-stoichiometric fashion may also contribute to the mechanism of the auxiliary binding sites^34–40^.

The direct binding pathway will be the predominant one (i.e., there will be no buffering effect) both at high ATP concentration (when *k*_*s*_ [ATP] ≫ k_tr_) and at very low [ATP] (when [ATP]≪K_w_ and the buffer sites are unoccupied). Hence, the importance of the buffering mechanism described here is in supporting rapid RecBCD activity in the middle range, ∼100–350 μM. This raises the question of its physiological role, as average ATP levels are in the millimolar range. Interestingly, recent works have shown that there is considerable variability between cells in a bacterial population, with a significant fraction of them exhibiting ATP levels much lower than the average^41^. The mechanism proposed here can serve to ensure the proper function of RecBCD, and hence proper damage repair, across the whole population. Finally, a dramatic decrease in intracellular ATP levels occurs upon exposure of cells to reactive oxygen species, such as those released by the host defense systems when it attempts to eliminate an invading bacterium^42^. Also, there is growing evidence for the correlation between DNA damage caused by oxidative stress and reduced intracellular ATP levels, and vice versa, in mammalian cells^43–45^. Such a correlation may also be a source of intracellular ATP depletion in bacterial cells. Although these species do not directly create DSBs, excessive oxidative stress can lead to DSBs via the conversion of unrepaired single-strand breaks. Hence, it is possible that the mechanism proposed here evolved as a defense mechanism, to support the activity of RecBCD during such damage-rich stress situations.

## Methods

### Reagents and purification of proteins

All chemicals and reagents were of the highest purity commercially available. ATP and ADP were purchased from Roche Molecular Biochemicals (Indianapolis, IN, USA). Adenosine 5′-(β,γ-imido)triphosphate (AMPpNp) was purchased from Sigma (St. Louis, MO, USA). A molar equivalent of MgCl_2_ was added to nucleotides immediately before use. Nucleotide concentrations were determined by absorbance using an extinction coefficient ε_259_ of 15,400 M^−1^ cm^−1^. The concentrations of *N*-methylanthraniloyl (mant) derivatives of ADP, 2′-deoxyADP, ATP, and 2′-deoxy ATP (Jena Bioscience, Jena, Germany) were determined using ε_255_ of 23,300 M^−1^ cm^−1^. Unless otherwise specified, all experiments were conducted in RecBCD Buffer (RB: 20 mM MOPS pH 7.4, 2 mM MgCl_2_,1 mM DTT, 0.1 mM EDTA and, unless specified, 75 NaCl). Over-expression and purification of recombinant RecBCD was based on the method described by Roman et. al.^46^. All steps of purification were carried out at 4 °C, and contained 20 mM MOPS pH 7.4, 2 mM MgCl_2_,1 mM DTT, 0.1 mM EDTA, 1 mM PMSF, 1 mM Benzamidine and the indicated salt concentration. Four liters of *E.coli* cells expressing RecBCD were lysed using Microfluidizer, followed by centrifugation at 10,000×*g*. The supernatant was further clarified by centrifugation at 100,000×*g* and treated with Benzonase for 2 h before initial purification by DEAE chromatography (weak anion exchanger to remove nucleic acids contaminants) using a linear NaCl gradient from 75 mM to 700 mM. RecBCD-containing DEAE fractions were eluted from a Q-sepharose column (strong anion exchanger which highly selects for active RecBCD) using a linear NaCl gradient from 75 mM to 1 M. Fractions containing RecBCD were precipitated using (NH_4_)_2_SO_4_ (45% saturation), and collected by centrifugation at 14,000×*g*. Precipitated RecBCD was resuspended and loaded onto Superdex 200 equilibrated with RB, as a final step of polishing and elution of RecBCD specifically from the monodisperse peak of the heterotrimer complex of RecBCD (Supplementary Fig. 15a, b). Fractions containing purified RecBCD were concentrated using an Amicon concentrator (50 kDa cutoff), aliquoted, and flash-frozen in liquid nitrogen before storage at −80 °C. RecBCD concentration was determined using an extinction coefficient ε_280_ of 4.2 × 10^5^ M^−1^ cm^−1^ in Guanidine chloride. To ensure RecBCD purity from nucleic acids, only protein fractions with 280/260 nm ratio >1.3 were used. RecBC and RecB^K29Q^C were purified using the same protocol and their concentrations were determined using ε_280_ of 3.7 × 10^5^ M^−1^ cm^−1^ in Guanidinium chloride. The stoichiometry of the purified complexes of RecBCD, RecBC, and RecB^K29Q^C was confirmed by polyacrylamide SDS-PAGE (Supplementary Fig. 15c).

The RecBC^mut^Ds were purified using the same protocol. The ε_280_ of the mutants was calculated using the Expasy ProtParam tool. The concentrations of RecBC^mut4^D and RecBC^mut7^D were determined using ε_280_ of 4.2 × 10^5^ M^−1^ cm^−1^; and for RecBC^mut13^D and RecBC^mut19^D using ε_280_ of 4.1 × 10^5^ M^−1^ cm^−1^ in Guanidinium chloride.

The expression of all the proteins were tested before the purification. About 10 ml of *E.coli* cells were incubated at 37 °C with 100 μg/ml Ampicillin, 34 μg/ml Chloramphenicol, and 10 μg/ml Spectinomycin. IPTG (1 mM) was added at OD_595_ = 0.5, and the cells were incubated for 5 h. To compare protein expression with or without IPTG induction, 1 ml of cell sample was taken before IPTG addition. The cells were lysed with 200 μl SDS-page sample buffer (X1), and a sample was electrophoresed on a polyacrylamide SDS-PAGE. From the gel, the stoichiometry of the three subunits of the complex was confirmed to be the same as the RecBCD WT (Supplementary Fig. 15d).

### Mant-nucleotide binding by Förster resonance energy transfer (FRET)

FRET measurements were performed with a PC1 spectrofluorometer (ISS, Champaign, IL), utilizing excitation and emission monochromators. The observation cell was regulated with a Peltier temperature controller at 25 ± 0.1 °C. All equilibrium binding reactions were performed in a 10 μl Precision cell fluorescence cuvette (Farmingdale, NY, USA), which allows minimal inner filter effects^47^ up to a concentration of ∼550 μM mant-Nucleotides. The experiments were conducted in RB, with 75 mM NaCl concentration. mant-Nucleotides were titrated with a 1:1 ratio to MgCl_2_. Equilibrium binding reactions of mant-Nucleotides to RecBCD were measured by FRET between RecBCD intrinsic tryptophan fluorescence (*λ*_ex_ = 280 nm) and bound mant-Nucleotide (fluorescence monitored at 90° through an emission monochromator at *λ*_em_ = 436 nm). We performed subtractions of background fluorescence of free nucleotides on the observed emission peak.

### Determination of equilibrium binding constants and thermodynamics parameters

Nucleotide-binding curves of the fluorescence change as a function of the free ligand concentration were fitted to the sum of two Hill equations:

1 $$y=p\cdot \frac{1}{1+{\left(\frac{{K}_{s}}{\left[{mN}\right]}\right)}^{{n}_{s}}}+\left(1-p\right)\cdot \frac{1}{1+{\left(\frac{{K}_{w}}{\left[{mN}\right]}\right)}^{{n}_{w}}}$$

where y is the fraction bound, *mN* is the ligand concentration, *K*_s_ and *K*_w_ are the dissociation constants of the first and second phase, respectively, *n*_*s*_ and *n*_*w*_ are the Hill coefficients of the first and second phase, respectively, and *p* is the partition coefficient (0 ≤ *p* ≤ 1). Of note, some uncertainty in the fitting parameters is introduced as a result of a finite overlap between the two phases in the isotherm. Moreover, the relation between the Hill coefficients and the number of sites is only tightly coupled in the case of infinitely high cooperativity. Hence, the measured coefficients were not used to derive the number of sites.

### Equilibrium dialysis measurements

Equilibrium dialysis measurements were performed with a Fast-Micro-Equilibrium Dialyzer™, with 25 μl chambers, and a Fast-Micro-Equilibrium Dialyzer 1 kDa MW cut-off membrane™ (Harvard Apparatus, Boston, MA, USA), in RB. Experiments were performed overnight at 4 °C. The concentration of free ligand was determined at the beginning of the experiment by measuring the absorbance at 259 nm (ε_ex,coeff_ = 15,400 M^−1^ cm^−1^, *T* = 25 °C), and then determined again at the end of incubation from the chamber of the free ligand only. Bound ligand concentration was determined using the mass conservation equation, [N]_i_ = 2[N]_f_ + n[RecBCD], where [N]_i_ is the concentration of the nucleotide at the beginning of the dialysis and [N]_f_ is the concentration at the end of the experiment, from the ligand-only chamber. In the high concentration regime, [RecBCD] was held at 45 μM and [ADP]_i_ or [AMPpNp]_i_ at 1 mM, with a 1:1 ratio to MgCl_2_. For the low concentration regime, we held [RecBCD] at 17 μM and nucleotides at 200 μM. We performed control experiments without RecBCD to estimate the time sufficient to reach equilibrium (overnight incubation) and to validate the level of nucleotide’s stickiness to the membrane (Supplementary Fig. 17). The total loss of nucleotides across the membrane was less than 0.5%.

### Molecular constructs for single-molecule experiments

To generate unwinding/translocation tracks of different lengths^18^, 600 and 4000 bp tracks were obtained using standard PCR reactions (Supplementary Table 11, IDT) and nicked using Nt.BbvCI for the Biotin-terminated track and Nb.BbvCI for the Digoxigenin-terminated one (enzymes from New England Biolabs), resulting in complementary 29-nucleotides flanked with three nucleotides (5′-TGC-3′). For the symmetric geometry, the 600 biotin and digoxigenin tracks were mixed at equal molar ratios for DNA annealing, creating a ∼1200 bp fragment. For the asymmetric geometries, 4000 bp tracks were annealed to complementary purchased oligonucleotides with the opposite modification (Supplementary Table 11, HPLC purified, IDT). This resulted in asymmetric tracks with 4000 bps and ∼35 nt single-stranded DNA on opposite sides. A ∼250 dsDNA stem containing the “601” sequence^48^ was amplified from a plasmid (a generous gift from Daniela Rhodes, MRC, Cambridge, UK) using primers listed in Supplementary Table 11, digested using DraIII-HF (New England Biolabs) overnight according to the manufacturer’s instructions, and ligated to the tracks.

### Optical tweezers

Experiments were performed in a custom-made double-trap optical tweezers apparatus^49^. Briefly, the beam from an 852 nm laser (TA PRO, Toptica) was coupled into a polarization-maintaining single-mode optical fiber. The collimated beam out of the fiber was split by a polarizing beam splitter (PBS) into two orthogonal polarizations, each directed into a mirror and combined again with a second BS. One of the mirrors is mounted on a nanometer scale mirror mount (Nano-MTA, Mad City Labs). An X2 telescope expands the beam and also images the plane of the mirrors into the back focal plane of the focusing microscope objective (Nikon, Plan Apo VC 60X, NA/1.2). Two optical traps are formed at the objective’s focal plane, each by a different polarization, and with a typical stiffness of 0.3–0.5 pN/nm. The light is collected by a second, identical objective, the two polarizations separated by a PBS, and imaged onto two Position Sensitive Detectors (PSDs, First Sensor). The position of the beads relative to the center of the trap is determined by back focal plane interferometry^50^. Calibration of the setup was done by the analysis of the thermal fluctuations of the trapped beads, which were sampled at 100 kHz.

### Single-molecule experiments

The complete construct was incubated for 15 min on ice with 0.9 µm polystyrene beads (Spherotech), coated with anti-Digoxigenin (anti-DIG). The reaction was then diluted 1000-fold in RB, with the addition of a 1:1 ratio of Mg·ATP, 0.05 mg/ml BSA, and an ATP regeneration system consisting of 7.5 mM phosphocreatine and 0.05 mg/ml creatine phosphokinase. Tether formation was performed in situ (inside the experimental chamber) by trapping an anti-DIG bead (bound by DNA) in one trap, trapping a 0.9 µm streptavidin-coated polystyrene beads in the second trap, and bringing the two beads into close proximity to allow binding of the biotin tag in the DNA to the streptavidin in the bead. The laminar flow cell (Lumicks) had four channels: streptavidin beads pre-bound to the DNA construct, anti-digoxigenin beads, RB, and RB with the addition of RecBCD. Single DNA tethers were verified in the buffer-only channel and then held at a tension of 5 pN and translocated to the RecBCD channel until activity was observed as indicated by a rapid decrease in the extension and increase in the force.

### Analysis of single-molecule experiments

Data were digitized at a sampling rate *f*_s_ = 2500 Hz using custom software written in LabView (National Instruments) and saved to a disk. All further processing of the data was done with Matlab (Mathworks). The measured extension was transformed into contour lengths (bps) using the worm-like chain model. Average velocities were calculated using the slope of a linear fit to 100-point smoothed contour lengths in the force ranges of 10–15 pN, where minimal force effect was probed on RecBCD’s rates.

### Steady-state ATPase activity

The steady-state ATPase activity of RecBCD (1 nM) was measured by following reduction in absorbance at 340 nm using the NADH-coupled assay at 25 ± 0.1 °C in RB, supplemented with saturating (300 nM) *E.coli*-DNA while varying the [Mg^2+^·ATP]. The [Mg^2+^·ATP] dependence of the steady-state ATPase rate was fitted to the quadratic form of the Briggs–Haldane equation:

2 $$v	={k}_{0}+\left({k}_{{{{{{{\mathrm{cat}}}}}}}}-{k}_{0}\right) \\ 	 \cdot \frac{\left({K}_{{{{{{\mathrm{m}}}}}}}+{\left[{{{{{{\mathrm{RecBCD}}}}}}}\right]}_{T}+{\left[{{{{{{\mathrm{ATP}}}}}}}\right]}_{T}\right)-\sqrt{{\left({K}_{{{{{{\mathrm{m}}}}}}}+{\left[{{{{{{\mathrm{RecBCD}}}}}}}\right]}_{T}+{\left[{{{{{{\mathrm{ATP}}}}}}}\right]}_{T}\right)}^{2}-4{\left[{{{{{{\mathrm{RecBCD}}}}}}}\right]}_{T}{\left[{{{{{{\mathrm{ATP}}}}}}}\right]}_{T}}}{2{\left[{{{{{{\mathrm{RecBCD}}}}}}}\right]}_{T}}$$

where *k*_0_ is the ATPase rate of RecBCD alone, *k*_cat_ is the turnover rate at saturating [ATP], *K*_m_ is the *apparent* Michaelis constant for substrate activation, [RecBCD]_*T*_ is the total RecBCD concentration, and [ATP]_*T*_ is the total concentration of ATP.

### Pre-steady state kinetic measurements

FRET between RecBCD intrinsic tryptophans (λ_ex_ = 280 nm) and mant-Nucleotides was monitored at 90° through a 400-nm long-pass colored-glass filter. For mant-nucleotide binding, photobleaching affected time courses beyond the fitting windows (>1 sec). Time course traces shown are averages of five to seven shots of 2000-points collected with the instrument in oversampling mode where the intrinsic time constant for data acquisition is ∼64 μs. Rapidly mixing the buffer with mant-nucleotide resulted in a linear dependence of the average fluorescence as a function of mant-nucleotides (Supplementary Fig. 18). Thus, data collected were globally fit to the equation $y\left(t,[{{{{{{\mathrm{mD}}}}}}}]\right)=\alpha \cdot \left[{{{{{{\mathrm{mD}}}}}}}\right]+\beta +{\sum }_{i=1}^{n}{A}_{i}\cdot (1-{{{{{\rm{exp}}}}}}(-{k}_{{{{{{{\mathrm{obs}}}}}}},i}\cdot t))$, where *α* and *β* describe the linear increase in the initial fluorescence due to increasing mantADP ([mD]), and *A*_*i*_ and *k*_obs*,i*_ represent the amplitude and the rate of the measured exponents. Parameter *n* was taken as 2 for normal conditions and as 1 in the presence of adenosine. The dead time of the instrument, determined from the reduction of 2,6-dicholorophenolindophenol with ascorbic acid in absorbance mode, is ∼1 msec. Fitting was limited to data beyond the measured dead time. The experiments were conducted in RB, and nucleotides were added in a 1:1 ratio mixture with MgCl_2_.

### DNA substrates for ensemble unwinding experiments

DNA oligonucleotides (Supplementary Table 12 and Supplementary Fig. 21) were purchased from IDT (Leuven, Belgium) and HPLC purified. The DNA substrates were obtained by folding or hybridization in 20 mM MOPS pH 7.4, 75 mM NaCl, 2 mM MgCl_2_, at 85 °C for 3 min followed by slow cooling to room temperature before storage at −20 °C. *E.coli* genomic DNA (Sigma) was digested with EcoRV and SnaBI (NEB) at 37 °C for 4 h to create blunt-end DNA substrates. DNA concentration was calculated by measuring absorbance at 260 nm and the number of moles of dsDNA was calculated according to *E.coli* genomic restriction map analysis.

### Fluorescence anisotropy monitoring of dsDNA unwinding by RecBCD

Fluorescence anisotropy unwinding time measurements were performed using a T-format excitation and emission module fitted on an SF-61DX2, TGK Scientific (Bradford on von, UK) stopped-flow apparatus thermostatted at 16.0 ± 0.1 °C. The concentrations stated are final after mixing. Samples were excited at λ_ex_ = 492 nm by using vertically polarized light. The emitted vertically and horizontally polarized light was monitored at 90° through a 515 nm long-pass colored-glass filter. The G-factor for correction of the differences in gain between the photomultiplier tube detectors was calculated as described by the instrument manufacturer. Data analysis of the time-resolved change in FA was performed according to Henn et al.^51^. Equilibrated mixtures of 1.1:1 complex RecBCD·hpDNA at 250 nM were rapidly mixed with equal volumes of 700 μM Mg·ATP (premixing). In addition, the ATP solution contained 20 μM of nonspecific ssDNA (25 mer) as a trap for RecBCD, and 5 μM of a nonfluorescent oligo as a trap for the released hairpin substrate after unwinding. All unwinding reactions were performed at 16 °C to allow sufficient time to monitor the unwinding lag phase. Shown time courses are averages of at least 13–15 transients. The time courses were fitted according to the following function:

3 $${FA}=\left\{\begin{array}{ll}A \hfill & ,{t}\le{t}_{{\mathrm lag},{\mathrm uw}} \\ \left(A-B\right)\,{{{{{\rm{exp }}}}}}[-{k}_{{{{{{{\mathrm{diss}}}}}}}}(t-{t}_{{{{{{{\mathrm{lag}}}}}}},{{{{{{\mathrm{uw}}}}}}}})]+B & ,t \, > \, {t}_{{{{{{{\mathrm{lag}}}}}}},{{{{{{\mathrm{uw}}}}}}}}\end{array}\right.$$

where *A, B* are the initial and final anisotropy values, *t*_*l*ag,uw_ is the lag-time corresponding to the unwinding duration, and *k*_diss_ is the dissociation constant of the oligo from the complex upon release. The fitting was done by minimizing the sum of the squared errors over the parameters {*A, B*, *k*_diss_, *t*_lag_} using the Nelder Mead Simplex method in MATLAB. Control experiments in the absence of ATP, and separately with a ssDNA substrate, confirmed the identification of the high and low FA signals, respectively (Supplementary Fig. 19).

### Fluorescence resonance energy transfer (FRET) monitoring of dsDNA unwinding by RecBCD

The DNA substrate for the FRET experiments is shown in Supplementary Table 12 and Supplementary Fig. 21. FRET between Cy3 (λ_ex_ = 550 nm) and Cy5 (λ_ex_ = 667 nm) fluorophores was monitored at 90° through a >650 nm long-pass colored-glass filter (Semrock). The measurements were performed using a TGK Scientific (Bradford on von, UK) stopped-flow apparatus (model: SF-61DX2) thermostatted at 16.0 ± 0.1 °C. The concentrations stated are final after mixing. Equilibrated mixtures of 1.1:1 complex RecBCD·hpDNA at 350 nM were rapidly mixed with equal volumes of 700 μM Mg·ATP (premixing). In addition, the ATP solution contained 20 μM of nonspecific ssDNA (25 mer) as a trap for RecBCD, and 5 μM of a nonfluorescent oligo as a trap for the released substrate after unwinding. All unwinding reactions were performed at 16 °C to allow sufficient time to monitor the unwinding lag phase. Shown time courses are averages of at least 8–10 transients. Control experiments separately with a ssDNA substrate confirmed the identification of the FRET signals (Supplementary Fig. 20).

### Detection of pauses in single-molecule experiments

Pausing analysis was done by applying a Chung–Kennedy nonlinear adaptive filter^52^, on the contour length vs. time data, with windows of size 25, 50, and 100 points and equal weights (Supplementary Fig. 22). The filtered data were processed with a pause detection threshold-based algorithm: peaks of the histogram of the smoothed contour dwell points including more than 50 points were suspected as pauses. To rule out false positives, we applied a series of thresholds on the pause durations (minimal pause 0.002 s), translocation times between pauses (minimal translocation time 0.001 s), and contour change between pauses (minimal contour difference 5 bp). Pause density was calculated as the ratio between the total number of pauses and the total translocated length for all the traces in a set.

### Structural modeling

Binding hot spots on RecBCD (1W36, [https://www.rcsb.org/structure/1w36]) were localized using the FTMap computational mapping server (http://ftmap.bu.edu/contact.php), based on 16 small organic molecules as probes (ethanol, isopropanol, isobutanol, acetone, acetaldehyde, dimethyl ether, cyclohexane, ethane, acetonitrile, urea, methylamine, phenol, benzaldehyde, benzene, acetamide, and *N*,*N* dimethylformamide)^53^.

### UV cross-linking experiment

Twenty micrograms of RecBCD were photoaffinity labeled^25^ with 600 μM of 8-azidoadenosine 5′-triphosphate and 8- azidoadenosine (Jena Bioscience), separately. The samples were incubated on ice for 30 min and then exposed for 2 min to 10 W of UV light (254 nm), using a UV Crosslinker instrument from Cleaver Scientific.

### Mass spectrometry experiment

Following UV cross-linking 2 μg of each sample was separated by SDS-PAGE using a NuPAGE^®^ Novex^®^ Bis-Tris Mini gel (4–12%) with NuPAGE^®^ MOPS SDS-Running buffer supplemented with 1x NuPAGE^®^ antioxidant (Life Technologies). After electrophoresis, the gel was stained with InstantBlue protein stain (Expedeon) and RecBCD bands were excised and subjected to in-gel trypsin digestion^54^. Alternatively, the entire crosslinked samples were denatured by incubation for 5 min at 95 °C, reduced with 5 mM DTT, alkylated with 12.5 mM chloroacetamide, and subjected to in-solution digestion with trypsin (1:100 w/w at 37 °C for 16 h). The resulting peptides were desalted using C18 Stagetips^55^ and resuspended in 2% acetonitrile, 0.1% formic acid.

The peptides were resolved by capillary chromatography and electrospray tandem mass spectrometry using a Q-Exactive-Plus mass spectrometer fitted with a capillary Ultimate 3000 RSLC nano-capillary UHPLC (Thermo-Fisher Scientific). The reversed-phase chromatographs were with about 30 cm long, 75-micron inner diameter capillary columns, home-packed with 3.5 m silica ReproSil-Pur C18-AQ resin (Dr. Maisch GmbH, Ammerbuch-Entringen, Germany)^56^. Peptides were eluted with a linear gradient of 5–28% acetonitrile in 0.1% formic acid. The capillary HPLC gradients were run at flow rates of 0.15 μl/min for 30 min. Data were acquired using a data-dependent “top 10” method, fragmenting the peptides by higher-energy collisional dissociation (HCD). Full scan MS spectra were acquired at a resolution of 70,000 at 200 m/z with a target value of 3 × 10^6^ ions and a maximum injection time of 20 msec. MS/MS ions were accumulated to an AGC target value of 10^5^ with a maximum injection time of 100 msec. Fragmentation was performed on charge states between 2 to 7. The peptide match option was set to Preferred. The normalized collision energy was set to 25% and MS/MS resolution was 17,500 at 200 m/z. Fragmented m/z values were dynamically excluded from further selection for 20 s.

### Mass spectrometry data analysis

The spectra of 8-Azido-ATP crosslinked samples was searched in Byonic^57^ v2.16.11 (Protein Metrics, San Carlos, CA). At first, a focused sequence database was generated by searching against *Escherichia coli* (strain K12) sequences downloaded from UniProt^58^ supplemented by known contaminant and decoy sequence and considered full tryptic peptides with up to three missed cleavages, while using 20 ppm mass tolerance for precursors and 20 ppm for fragment ions. Carbamidomethylated cysteine was set as a fixed modification, methionine oxidation was set as variable modification, and the FDR was set to 1%. The generated focused database was used for a wildcard search that considers all possible mass deltas between the candidate peptide mass and the mass of the precursor ion. The handful ATP binding site mapping that were based on mass spectrometry studies of that 8-Azido-ATP cross-linking indicated that the expected mass shift upon 8-Azido-ATP modification is around 520 Da^59^. To also consider the possibility of ATP hydrolysis during the cross-linking experiment and MS sample preparation the wildcard range was set to 0 to +540 Da.

The spectra of 8-Azido-Adenosine was performed using a similar approach, except that the wildcard search was conducted using a range of 0 to +320 Da due to the smaller mass of the crosslinker. These search results were validated by additional MS/MS analysis in which peak lists obtained from MS/MS spectra were identified using X! Tandem version X! Tandem Vengeance (2015.12.15.2)^60^, MS Amanda version 2.0.0.9695^61^, MS-GF+ version Release (v2018.04.09)^62^, Comet version 2018.01 rev.0^63^, and Tide version unknown^64^. The search was conducted using SearchGUI version [v3.3.3]^65^. Protein identification was conducted against a concatenated target/decoy^66^ version of the *E.coli* (strain K12) from UniProtKB as indicated above. The decoy sequences were created by reversing the target sequences in SearchGUI. The identification settings were as follows: Trypsin, Specific, with a maximum of two missed cleavages 20.0 ppm as MS1 and 20.0 ppm as MS2 tolerances; fixed modifications: carbamidomethylation of cysteine; variable modifications: oxidation of methionine, deamidation of asparagine, deamidation of glutamine, and AzidoAdenosine of leucine/isoleucine/tyrosine/threonine or serine. Several options for mass shifts caused by AzidoAdenosine modification were tested: +277.0685, +278.0764, +279.0842, and +280.0920 Da. Peptides and proteins were inferred from the spectrum identification results using PeptideShaker version 1.16.27^67^. Peptide Spectrum Matches (PSMs), peptides and proteins were validated at a 1.0% false discovery rate (FDR) estimated using the decoy hit distribution. Posttranslational modification localizations were scored using the *D*-score^68^ and the phosphoRS score^69^ with a threshold of 95.0 as implemented in the compomics-utilities package^70^. The peptide sequences with specific mass shifts corresponding to various 8-Azidoadenosine modifications were ranked based on their phosphoRS scores. The mass spectrometry data along with the identification results have been deposited to the ProteomeXchange Consortium^71^ via the PRIDE partner repository^72^ with the dataset identifier PXD031540.

### Recognition of possible ATP binding sites from mass spectrometry analysis

To further scrutinize and identify the peptide sequences which possibly contain the auxiliary ATP binding sites, we mapped the peptides onto the FTmap of the RecC subunit. Peptide sequences structurally overlapping with one or more small molecule clusters were selected, with the rationale that ATP or adenosine molecules would also have a high probability of binding to the peptides in these regions. Consequently, we identified specific amino acids on these peptides interacting or in-contact with the clusters, from both the 8-Azidoadenosine and the 8-AzidoATP samples, with some common to both. This analysis resulted in the identification of four putative ATP/Adenosine-binding peptide sequences.

### Basis of point mutations

Point mutations were designed on 19 amino acids from the four putative ATP/Adenosine-binding peptide sequences. The basis for the point mutations was to substitute the existing amino acid with another which has less relative volume and in some cases also by changing the charge or hydrophobicity. Various combinations of these 19 point mutations were introduced into four different mutants.

### Preparation of mutant plasmids

The molecular biology strategy to incorporate the mutations in RecC, in order to create the various accumulative mutants are as described below. For RecBC^mut4^D, the mutations were introduced to a *recC* gene-containing plasmid (pPB520), which contains a Chloramphenicol (Cm) resistance gene via site-directed mutagenesis (SDM). Primer sets for each mutagenesis were designed using the software at https://nebasechanger.neb.com. Before the PCR reaction, primers were phosphorylated at the 5′ end. The PCR reaction tubes were incubated for 1 h at 37 °C with DpnI endonuclease (to digest the methylated parental DNA template and to select mutation-containing synthesized DNA). Finally, the plasmids were ligated using T4 DNA ligase (New England Biolabs) by incubation for 1 h at room temperature. We prepared four mutations for RecBC^mut4^D mutants. All the mutations were a single amino acid substitution.

For preparing the plasmid for RecBC^mut19^D, a DNA sequence corresponding to part of the *recC* gene with the 19 point mutations incorporated, was synthesized and purchased from Bio Basic Canada Inc, in the pUC57 vector. The insert was extracted from the pUC57 vector by using restriction enzymes BamHI and SaII and ligated to plasmid pPB520, in the appropriate region of the *recC* gene. For preparing the plasmid for RecBC^mut7^D and RecBC^mut13^D, the pPB520 plasmid with 19 point mutations was cut by restriction enzymes PvuI, MluI, and BsiWI respectively. The plasmid containing the *recC* gene (pPB520) was also cut with these combinations of restriction enzymes. The corresponding mutated plasmid sequences were incorporated into the appropriate regions of pPB520 by ligation. The mutated plasmids were confirmed by sequencing and transformed (electroporation method) along with the pPB800 plasmid (containing *recB* and *recD*) into the V330 null strain.

### Global fitting to the model

To perform a global fitting to the scheme in Fig. 6c, we calculated its binding partition function or binding polynomial^17^, as:

$$\Psi \left(T\right)= \,1+\left({K}_{{{{{{\mathrm{s}}}}}}}+{K}_{{{{{{\mathrm{w}}}}}}}\right)T+\left({K}_{{{{{{\mathrm{s}}}}}}}^{2}+{K}_{{{{{{\mathrm{s}}}}}}}{K{{{{{\mathrm{w}}}}}}}+a{K}_{{{{{{\mathrm{w}}}}}}}^{2}\right){T}^{2}+\left({K}_{{{{{{\mathrm{s}}}}}}}^{2}{K}_{{{{{{\mathrm{w}}}}}}}+a{K}_{{{{{{\mathrm{s}}}}}}}{K}_{{{{{{\mathrm{w}}}}}}}^{2}+{a}^{2}{K}_{{{{{{\mathrm{w}}}}}}}^{3}\right){T}^{3}\\ +\left({aK}{{{{{{\mathrm{s}}}}}}}^{2}{K}_{{{{{{\mathrm{w}}}}}}}^{2}+a{K}_{{{{{{\mathrm{s}}}}}}}{K}_{{{{{{\mathrm{w}}}}}}}^{3}+{a}^{3}{K}_{{{{{{\mathrm{w}}}}}}}^{4}\right){T}^{4}+\left({a}^{2}{K}_{{{{{{\mathrm{s}}}}}}}{K}_{{{{{{\mathrm{w}}}}}}}^{3}+{a}^{3}{K}_{{{{{{\mathrm{s}}}}}}}{K}_{{{{{{\mathrm{w}}}}}}}^{4}\right){T}^{5}+{a}^{3}{K}_{{{{{{\mathrm{s}}}}}}}^{2}{K}_{{{{{{\mathrm{w}}}}}}}^{4}{T}^{6}$$

where *K*_s_*,K*_w_ denote the binding association constants (${{K}_{i}=k}_{i}^{+}/{k}_{i}^{-}$) for the strong and weak sites, respectively, *a* the cooperativity constant, and T the ATP concentration. The association constants were calculated as ${k}_{i}^{+}/{k}_{i}^{-}$ for the strong and weak sites. The fraction of ATP bound is then given by $\frac{\partial {{{{{\rm{ln}}}}}}\Psi }{\partial {ln}T}$, to which our data was fitted. Simulations of mant-nucleotide-binding kinetics were generated by numerically solving the system of differential equations describing the model for up to 30 msec. The time course of each of the bound states was weighted by the number of bound molecules and the overall weighted sum was fitted to double exponentials. Simulations of DNA unwinding by RecBCD were generated by assuming a direct proportionality with hydrolysis, and numerically solving the resulting system of differential equations resulting from the model, including the catalysis steps, up to 30 s. A linear fit to these results was used to calculate the unwinding rates. The sum of the normalized squared error for all experiments, adjusted for their temperature using the temperature-dependent ATPase rates in Supplementary Fig. 4B, was minimized using the global search tool (scatter search algorithm with constrained optimization, interior point) in MATLAB for the parameter set describing: $\{{k}_{{{{{{\mathrm{s}}}}}}}^{+},{k}_{{{{{{\mathrm{s}}}}}}}^{-},{k}_{{{{{{\mathrm{w}}}}}}}^{+},{k}_{{{{{{\mathrm{w}}}}}}}^{-},{k}_{{{{{{{\mathrm{tr}}}}}}}}^{+},{k}_{{{{{{{\mathrm{tr}}}}}}}}^{-},a,{v}_{{{{{{\rm{max }}}}}}}\}$, where ${k}_{w}^{+}$ was taken as a nonnegative linear decreasing function of adenosine (${k}_{{{{{{\mathrm{w}}}}}}}^{+}=\alpha \left[{{{{{{\mathrm{Adenosine}}}}}}}\right]+\beta$), and ${k}_{{{{{{\mathrm{w}}}}}}}^{-}$ and ${k}_{{{{{{{\mathrm{tr}}}}}}}}^{+}$ were taken as nonnegative linear decreasing functions of [NaCl] (${k}_{{{{{{\mathrm{w}}}}}}}^{-}=\gamma \,\left[{{{{{{\mathrm{NaCl}}}}}}}\right]+\delta$, ${k}_{{{{{{{\mathrm{tr}}}}}}}}^{+}=\epsilon \left[{{{{{{\mathrm{NaCl}}}}}}}\right]+\kappa$), resulting in a global fitting of 11 parameters.

The number of weak binding sites included in the model was based on the equilibrium binding curves: We developed the equations of kinetic schemes similar to the ones in Fig. 6c, where the number of catalytic and non-catalytic sites were varied. For all these cases, the model was globally fitted to the data, and the resulting (calculated) binding curves were compared to the experimental data. We found that four auxiliary sites, in addition to the two catalytic sites, produced the best fitting to our measured data. Increasing the number of weak sites reduces the amplitudes of the catalytic sites, whilst schemes with a smaller number of sites fail to capture the cooperatively measured.

To test the robustness of the model, we applied a bootstrap Monte Carlo method. We generated 100 random data sets, drawing each parameter from a distribution with a mean equal to the experimentally measured value, and a width equal to the experimental uncertainty. We then fitted each dataset to the model using a global search algorithm, resulting in 100 sets of fitting parameters. Histograms of each of the fitted parameters were then fit to Gaussian functions, as shown in Supplementary Fig. 23. This analysis shows that the model robustly converges to a global minimum.

### Survival probability assay

Bacterial strains in 50% glycerol were isolated by streaking on selective agar plates containing 100 μg/ml Ampicillin, 34 μg/ml Chloramphenicol, and 10 μg/ml Spectinomycin (except for the null strain). In the following stages, all the bacterial strains (except for the null strain) were grown in selective LB broth (100 μg/ml Ampicillin, 34 μg/ml Chloramphenicol, and 10 μg/ml Spectinomycin). A single colony was inoculated into 5 ml starters and incubated overnight at 37 °C with moderate shaking of 220 rpm. A sample from each starter was diluted (x4), and the OD_600_ was measured (Ultrospec 10 Cell Density Meter). About 50 ml of selective LB broth was inoculated according to the calculated OD_600_. The cells were cultured in a shaker incubator at 37 °C, 220 rpm. When the OD_600_ reached 0.5 (mid-log phase), IPTG was added to a final concentration of 1 mM, and the cells were incubated for an additional hour. A series of dilutions were prepared (10^−2^–10^−5^), and 100 μl from each diluted subculture were loaded by spread plating on separate warm agar plates containing 10 mM IPTG (Supplementary Fig. 10). The plates were left at room temperature for 30 min before UV irradiations. Three to eight plates were made for each UV dose (10 and 20 J/m^2^). Dilutions in the 10^−2^–10^−5^ range were used for the control plates of 0 and for 10 J/m^2^, and 10^−2^–10^−5^ dilutions were used for 20 J/m^2^. The plates designated for UV irradiations were placed inside a 254 nm UV irradiation chamber (Vilber Lourmat, Courtesy of the Schuster Lab). After UV treatment, the plates were incubated overnight in the dark at 37 °C. The plates were photographed, and the colonies were counted via OpenCFU software to determine the number of colony-forming units per milliliter (CFU/mL). The survival percentage was calculated (for each experiment separately) by dividing the mean number of colonies obtained in each UV dosage by the control plates (0 J/m^2^).

### Sequence conservation analysis

The evolutionary conservation analysis for sequences and amino acids were performed using an online bioinformatics tool, ConSurf server (https://consurf.tau.ac.il/)^73–75^. The analysis was performed with one iteration using the HMMER homolog search algorithm, with an E-value cutoff of 0.0001 on the UniProt protein database. The analysis was done by sampling 150 homologous sequences with the reference sequence, which were chosen with a maximal %ID of 95 and minimal %ID of 35 between the homologs. The reference sequence was RecC from *E.coli*, Uniprot ID PO7648. The nine-color conservation scores are projected onto the 3D structure of RecC by ConSurf^76,77^ and the colored protein structure is shown using PyMol.

### Reporting summary

Further information on research design is available in the Nature Research Reporting Summary linked to this article.

## Supplementary information

Supplementary Information

Reporting Summary

## Data Availability

The data that support this study are available from the corresponding author upon reasonable request. Mass spectrometry generated in this study has been deposited in the PRIDE partner repository with the dataset identifier PXD031540. Source data are provided with this paper.

## Source data

Source Data

## References

[1] Smith GR (2012). How RecBCD enzyme and Chi promote DNA break repair and recombination: a molecular biologist’s View. Microbiol. Mol. Biol. Rev..

[2] Wiktor J, van der Does M, Büller L, Sherratt DJ, Dekker C (2018). Direct observation of end resection by RecBCD during double-stranded DNA break repair in vivo. Nucleic Acids Res..

[3] Boehmers, P. E. & Emmersonq, P. T. The RecB subunit Escherichia coli RecBCD enzyme couples ATP hydrolysis to DNA unwinding. *J Biol Chem*. **267**, 4981–4987 (1992). 1311326

[4] Dillingham MS, Spies M, Kowalczykowski SC (2003). RecBCD enzyme is a bipolar DNA helicase. Nature.

[5] Singleton MR, Dillingham MS, Gaudier M, Kowalczykowski SC, Wigley DB (2004). Crystal structure of RecBCD enzyme reveals a machine for processing DNA breaks. Nature.

[6] Masterson C (1992). Reconstitution of the activities of the RecBCD holoenzyme of *Escherichia coli* from the purified subunits. J. Biol. Chem..

[7] Taylor AF, Smith GR (1995). Strand specificity of nicking of DNA at Chi sites by RecBCD enzyme: modulation by ATP and magnesium levels. J. Biol. Chem..

[8] Taylor AF, Smith GR (2003). RecBCD enzyme is a DNA helicase with fast and slow motors of opposite polarity. Nature.

[9] Dillingham MS, Kowalczykowski SC (2008). RecBCD enzyme and the repair of double-stranded DNA breaks. Microbiol. Mol. Biol. Rev..

[10] Yeeles JTP, Dillingham MS (2010). The processing of double-stranded DNA breaks for recombinational repair by helicase–nuclease complexes. DNA Repair..

[11] Wu CG, Bradford C, Lohman TM (2010). *Escherichia coli* RecBC helicase has two translocase activities controlled by a single ATPase motor. Nat. Struct. Mol. Biol..

[12] Xie F, Wu CG, Weiland E, Lohman TM (2013). Asymmetric regulation of bipolar single-stranded DNA translocation by the two motors within *Escherichia coli* RecBCD helicase. J. Biol. Chem..

[13] Lohman, T. M. & Fazio, N. T. How Does a Helicase Unwind DNA? Insights from RecBCD Helicase. *BioEssays* **40**, 1–6 (2018). 10.1002/bies.201800009 PMC6154392 29603305

[14] Lucius AL (2002). DNA unwinding step-size of E. coli RecBCD helicase determined from single turnover chemical quenched-flow kinetic studies. J. Mol. Biol..

[15] Lucius AL, Maluf NK, Fischer CJ, Lohman TM (2003). General methods for analysis of sequential ‘n-step’ kinetic mechanisms: application to single turnover kinetics of helicase-catalyzed DNA unwinding. Biophys. J..

[16] Lucius AL, Jason Wong C, Lohman TM (2004). Fluorescence stopped-flow studies of single turnover kinetics of E.coli RecBCD helicase-catalyzed DNA unwinding. J. Mol. Biol..

[17] Wu, C. G. & Lohman, T. M. Influence of DNA end structure on the mechanism of initiation of DNA unwinding by the *Escherichia coli* RecBCD and RecBC helicases. **382**, 312–326 (2008). 10.1016/j.jmb.2008.07.012 PMC3174691 18656489

[18] Zananiri R (2019). Synergy between RecBCD subunits is essential for efficient DNA unwinding. Elife.

[19] Liu B, Baskin RJ, Kowalczykowski SC (2013). DNA unwinding heterogeneity by RecBCD results from static molecules able to equilibrate. Nature.

[20] Korangy1, F. & Julin, D. A. Efficiency of ATP hydrolysis and DNA unwinding by the RecBC enzyme from *Escherichia coli*. *Biochemistry* **33**, 9552 (1994). 10.1021/bi00198a022 8068630

[21] Roman LJ, Kowalczykowski SC (1989). Characterization of the helicase activity of the *Escherichia coli* RecBCD enzyme using a novel helicase assay. Biochemistry.

[22] Zangi R, Hagen M, Berne BJ (2007). Effect of ions on the hydrophobic interaction between two plates. J. Am. Chem. Soc..

[23] Bianco PR (2001). Processive tanslocation and DNA unwinding by individual RecBCD enzyme molecules. Nature.

[24] KJ M, TM L (1994). Kinetic mechanism of adenine nucleotide binding to and hydrolysis by the *Escherichia coli* Rep monomer. 1. Use of fluorescent nucleotide analogues. Biochemistry.

[25] Julin DA, Lehman IR (1987). Photoaffinity labeling of the recBCD enzyme of *Escherichia coli* with 8-azidoadenosine 5’-triphosphate. J. Biol. Chem..

[26] Amundsen, S. K., Taylor, A. F. & Smith, G. R. Chi hotspot control of RecBCD helicase-nuclease by long-range intramolecular signaling. *Sci. Rep*. **10**, 19415 (2020). 10.1038/s41598-020-73078-0 PMC7644769 33154402

[27] Bonura T, Smith KC (1975). Quantitative evidence for enzymatically-induced DNA double-strand breaks as lethal lesions in uv irradiated pol ^+^ and polal strains of *E. coli* K-12. Photochem. Photobiol..

[28] Smith KC, Wang T-CV, Sharma RC (1987). recA-dependent DNA repair in UV-irradiated *Escherichia coli*. J. Photochem. Photobiol. B Biol..

[29] Lucius AL, Jezewska MJ, Bujalowski W (2006). The *Escherichia coli* PriA Helicase has two nucleotide-binding sites differing dramatically in their affinities for nucleotide cofactors. 1. Intrinsic affinities, cooperativities, and base specificity of nucleotide cofactor binding. Biochemistry.

[30] Lucius AL, Jezewska MJ, Bujalowski W (2006). Allosteric interactions between the nucleotide-binding sites and the ssDNA-binding site in the PriA helicase-ssDNA complex. 3. Biochemistry.

[31] Akabayov SR, Akabayov B, Wagner G (2014). Human translation initiation factor eIF4G1 possesses a low-affinity ATP binding site facing the ATP-binding cleft of eIF4A in the eIF4G/eIF4A complex. Biochemistry.

[32] Ušaj M, Moretto L, Vemula V, Salhotra A, Månsson A (2021). Single molecule turnover of fluorescent ATP by myosin and actomyosin unveil elusive enzymatic mechanisms. Commun. Biol..

[33] Absmeier E (2021). Long-range allostery mediates cooperative adenine nucleotide binding by the Ski2-like RNA helicase Brr2. J. Biol. Chem..

[34] Patel A (2017). Biochemistry: ATP as a biological hydrotrope. Science.

[35] Ou, X. et al. ATP Can Efficiently Stabilize Protein through a Unique Mechanism. *JACS Au*. **1**, 1766–1777 (2021). 10.1021/jacsau.1c00316 PMC8549052 34723279

[36] Sridharan S (2019). Proteome-wide solubility and thermal stability profiling reveals distinct regulatory roles for ATP. Nat. Commun..

[37] Nishizawa M (2021). Effects of Weak Nonspecific Interactions with ATP on Proteins. J. Am. Chem. Soc..

[38] Mehringer J (2021). Hofmeister versus Neuberg: is ATP really a biological hydrotrope?. Cell Rep. Phys. Sci..

[39] Sarkar S, Mondal J (2021). Mechanistic insights on ATP’s role as a hydrotrope. J. Phys. Chem. B.

[40] Song J (2021). Adenosine triphosphate energy-independently controls protein homeostasis with unique structure and diverse mechanisms. Protein Sci..

[41] Yaginuma H (2015). Diversity in ATP concentrations in a single bacterial cell population revealed by quantitative single-cell imaging. Sci. Rep..

[42] Winter J, Linke K, Jatzek A, Jakob U (2005). Severe oxidative stress causes inactivation of DnaK and activation of the redox-regulated chaperone Hsp33. Mol. Cell.

[43] Murata MM (2019). NAD+ consumption by PARP1 in response to DNA damage triggers metabolic shift critical for damaged cell survival. Mol. Biol. Cell.

[44] Jha B, Pohlit W (1993). Reversibility of inhibition of DNA double strand break repair by 2-deoxy-d-glucose in ehrlich ascites tumour cells. Int. J. Radiat. Biol..

[45] Schütt F, Aretz S, Auffarth GU, Kopitz J (2012). Moderately reduced ATP levels promote oxidative stress and debilitate autophagic and phagocytic capacities in human RPE cells. Investig. Ophthalmol. Vis. Sci..

[46] Roman LJ, Kowalczykowski SC (1989). Characterization of the adenosinetriphosphatase activity of the *Escherichia coli* RecBCD enzyme: relationship of ATP hydrolysis to the unwinding of duplex DNA. Biochemistry.

[47] Birdsall B (1983). Correction for light absorption in fluorescence studies of protein-ligand interactions. Anal. Biochem..

[48] Lowary PT, Widom J (1998). New DNA sequence rules for high affinity binding to histone octamer and sequence-directed nucleosome positioning. J. Mol. Biol..

[49] Rudnizky S (2016). H2A.Z controls the stability and mobility of nucleosomes to regulate expression of the LH genes. Nat. Commun..

[50] Gittes F, Schmidt CF (1998). Interference model for back-focal-plane displacement detection in optical tweezers. Opt. Lett..

[51] Henn A (2010). Pathway of ATP utilization and duplex rRNA unwinding by the DEAD-box helicase, DbpA. Proc. Natl Acad. Sci. USA.

[52] Chung SH, Kennedy RA (1991). Forward-backward non-linear filtering technique for extracting small biological signals from noise. J. Neurosci. Methods.

[53] Kozakov D (2015). The FTMap family of web servers for determining and characterizing ligand-binding hot spots of proteins. Nat. Protoc..

[54] Ziv, I. et al. A perturbed ubiquitin landscape distinguishes between ubiquitin in trafficking and in proteolysis. *Mol. Cell. Proteom*. **10**, M111.009753 (2011). 10.1074/mcp.M111.009753 PMC3098606 21427232

[55] Rappsilber J, Ishihama Y, Mann M (2003). Stop and go extraction tips for matrix-assisted laser desorption/ionization, nanoelectrospray, and LC/MS sample pretreatment in proteomics. Anal. Chem..

[56] Ishihama, Y., Rappsilber, J., Andersen, J. S. & Mann, M. Microcolumns with self-assembled particle frits for proteomics. *J. Chromatogr. A* **979**, 233–239 (2002). 10.1016/s0021-9673(02)01402-4 12498253

[57] Bern, M., Kil, Y. J. & Becker, C. Byonic: advanced peptide and protein identification software. *Curr. Protoc. Bioinforma*. **Chapter 13**, (2012). 10.1002/0471250953.bi1320s40 PMC3545648 23255153

[58] Bateman A (2021). UniProt: the universal protein knowledgebase in 2021. Nucleic Acids Res..

[59] Ahnert F, Schmid R, Altendorf K, Greie JC (2006). ATP binding properties of the soluble part of the KdpC subunit from the *Escherichia coli* K+-transporting KdpFABC P-Type ATPase. Biochemistry.

[60] Craig R, Beavis RC (2004). TANDEM: Matching proteins with tandem mass spectra. Bioinformatics.

[61] Dorfer V (2014). MS Amanda, a universal identification algorithm optimized for high accuracy tandem mass spectra. J. Proteome Res..

[62] Kim S, Pevzner PA (2014). MS-GF+ makes progress towards a universal database search tool for proteomics. Nat. Commun..

[63] Eng JK, Jahan TA, Hoopmann MR (2013). Comet: an open-source MS/MS sequence database search tool. Proteomics.

[64] Diament BJ, Noble WS (2011). Faster SEQUEST searching for peptide identification from tandem mass spectra. J. Proteome Res..

[65] Vaudel M, Barsnes H, Berven FS, Sickmann A, Martens L (2011). SearchGUI: an open-source graphical user interface for simultaneous OMSSA and X!Tandem searches. Proteomics.

[66] Elias JE, Gygi SP (2010). Target-decoy search strategy for mass spectrometry-based proteomics. Methods Mol. Biol..

[67] Vaudel M (2015). PeptideShaker enables reanalysis of MS-derived proteomics data sets: to the editor. Nat. Biotechnol..

[68] Vaudel M (2013). D-score: a search engine independent MD-score. Proteomics.

[69] Taus T (2011). Universal and confident phosphorylation site localization using phosphoRS. J. Proteome Res..

[70] Barsnes H (2011). Compomics-utilities: an open-source Java library for computational proteomics. BMC Bioinforma..

[71] Vizcaíno JA (2014). ProteomeXchange provides globally coordinated proteomics data submission and dissemination. Nat. Biotechnol..

[72] Martens L (2005). PRIDE: the proteomics identifications database. Proteomics.

[73] Ashkenazy H, Erez E, Martz E, Pupko T, Ben-Tal N (2010). ConSurf 2010: calculating evolutionary conservation in sequence and structure of proteins and nucleic acids. Nucleic Acids Res..

[74] Celniker G (2013). ConSurf: using evolutionary data to raise testable hypotheses about protein function. Isr. J. Chem..

[75] Ashkenazy H (2016). ConSurf 2016: an improved methodology to estimate and visualize evolutionary conservation in macromolecules. Nucleic Acids Res..

[76] Glaser F (2003). ConSurf: identification of functional regions in proteins by surface-mapping of phylogenetic information. Bioinformatics.

[77] Landau M (2005). ConSurf 2005: the projection of evolutionary conservation scores of residues on protein structures. Nucleic Acids Res..

