# Supplementary material for: Auxiliary ATP binding sites support DNA unwinding by RecBCD
Source: Nat Commun. 2022 Apr 4;13:1806. doi: 10.1038/s41467-022-29387-1 (PMC8980037; doi:10.1038/s41467-022-29387-1)
Supplement: Supplementary file 1 — Supplementary Information [file 41467_2022_29387_MOESM1_ESM.pdf]

Supplementary Information

SUPPLEMENTARY NOTE 1

Nucleotide binding to RecBCD cannot be accounted by the two catalytic sites

Binding of mant-nucleotides to RecBCD and RecBCD·DNA measured by FRET exhibited a biphasic binding pattern. Since the RecBCD complex possesses two well defined catalytic nucleotide-binding sites, in RecB and RecD, one might postulate that these two binding phases reflect binding of nucleotides to each one of these catalytic sites, or that a cooperative model of two binding sites can give rise to such pattern. However, here we show that the biphasic nature of the binding isotherm cannot be accounted for by two binding sites. In what follows we analyze the possible cases involving two binding sites and show that none of them can give rise to a biphasic curve.

Model 1: Two independent binding sites. In this case, each site represents binding of a nucleotide to a catalytic site and the sites are non-cooperative in their binding. It is well known that binding of a single ligand to a single site gives rise to a hyperbolic pattern. Hence, the overall occupancy, y, can be expressed as a weighted sum of two hyperbolas, i.e.

$$y = p\frac{x}{x + K_1} + (1 - p)\frac{x}{x + K_2} \qquad \text{(Supplementary Eq. 1)}$$

where x is the ligand concentration and K_1 and K_2 the dissociation constants of the binding sites. The derivative of this curve,

$$\frac{\partial}{\partial x}y = \frac{pK_1}{(x + K_1)^2} + \frac{(1 - p)K_2}{(x + K_2)^2} \qquad \text{(Supplementary Eq. 2)}$$

is always positive and decreases with increasing concentrations. Therefore, y will always have a hyperbolic pattern regardless of the values of K_1 and K_2. Simply put, the sum of two concave hyperbolas is also a concave function (Supplementary Fig. 2A). Since for a biphasic pattern, the derivative of the curve should decrease in the first phase and then increase in the second, this model cannot give rise to a biphasic pattern.

Model 2: A Hill model for two cooperative binding sites. The Hill equation,

$$y = \frac{x^n}{x^n + K^n} \qquad \text{(Supplementary Eq. 3)}$$

is the most common way to describe cooperativity. Two cooperative sites on a macromolecule will result in a Hill coefficient that is non-unity. For positive cooperativity, the Hill coefficient is greater

than unity resulting in a sigmoidal pattern which cannot explain the data (Supplementary Fig. 2B). For negative cooperativity, the Hill coefficient is lower than unity resulting in a derivative,

$$\frac{\partial}{\partial x} y = \frac{nK^n x^{n-1}}{(x^n + K^n)^2} \quad \text{(Supplementary Eq. 4)}$$

that is always positive and decreasing with increasing ligand concentration, resulting in a hyperbolic-like curve (Supplementary Fig. 2C). Hence, this model cannot explain our data.

Thus, two sites are insufficient to give rise to a biphasic binding pattern. Following this argument, one can decompose the binding curve into two phases, one hyperbolic with high affinity and one sigmoidal with weak binding. The decomposition is shown in Supplementary Fig. 2D. The first hyperbolic phase could be due to a single site, or multiple loosely coupled binding sites. The second binding phase represents a sigmoidal pattern, necessitating additional sites (at least two, and cooperative) that are distinct from the site/s giving rise to the first phase. Assuming that the affinities of the weak and strong sites are well separated, we phenomenologically describe the total occupancy by the weighted sum of two Hill equations:

$$y = p \frac{x^{n_1}}{x^{n_1} + K_s^{n_1}} + (1 - p) \frac{x^{n_2}}{x^{n_2} + K_w^{n_2}} \quad \text{(Supplementary Eq. 5)}$$

where (K_s, K_w) are the microscopic dissociation constants, and (n_s, n_w) the Hill coefficients of the strong and weak phases, respectively. As shown in Supplementary Fig. 2D, this model can result in a biphasic pattern. Figs. 1A, B and D show that it fits our data well.

SUPPLEMENTARY NOTE 2

mant-Nucleotides binding kinetics detailed analysis

Time courses of mantADP (Fig. 3A) and mantATP (Supplementary Fig. 6) binding to RecBCD displayed a double-exponential pattern. The fast phase observed rate $\left(k_{obs}^{fast}\right)$ of mantATP and mantADP binding to RecBCD and RecBCD·DNA complexes exhibited a hyperbolic concentration dependence on mant-nucleotide (mN) concentration (Fig. 3B and Supplementary Fig. 6). Hence, it is modeled by a reaction mechanism where a rapid equilibrium step is followed by an isomerization step to a high fluorescence complex $RecBCD{\cdot}mN^*$, as follows:

$$RecBCD + mN \xleftrightarrow{K_1} RecBCD \cdot mN \xleftrightarrow{k_{+2}} RecBCD \cdot mN^*$$

This two-step binding reaction predicts that the observed rate constants for mN binding should obey a rectangular hyperbola according to:

$$k_{obs} = \frac{k_{+2}[mN]}{K_1 + [mN]} + k_{-2} \qquad \text{(Supplementary Eq. 6)}$$

where K_1 is a measure of the strength of the collision complex between RecBCD and mN, and $k_{\pm 2}$ are the on (+) and off (-) rates of isomerization. Supplementary Table 6 summarizes the rate constants of mant-nucleotide binding to RecBCD and RecBCD·DNA complexes. The kinetics of both mantATP and mantADP binding to RecBCD are extremely fast with a relatively tight collision complex: K_1 is in the ranges of tenths of μM in all cases. Additionally, the overall apparent second order binding constants, k_{+2}/K_1, are an order of magnitude faster than many other molecular motors studied [1,2]. For comparison, it is ~30 fold faster than Rep; a SF1 DNA helicase [3]. Remarkably, the maximum rate of the observed isomerization is faster than 1000 s^{-1} at 6° C supporting rapid kinetics of nucleotide binding to RecBCD and RecBCD·DNA complexes. Binding of mantATP to RecBCD or RecBCD·DNA is irreversible, with an intercept being indistinguishable from the origin ($k_{-2} \approx 0$).

The binding kinetics for mantATP and mantADP, in the absence or presence of a DNA substrate, is overall very similar in its mechanism and rates (Supplementary Fig. 9 & 10 and Supplementary Table 6). This may suggest that RecBCD has an overall weak selectivity for the type of nucleotide. The structural implication may be that RecBCD nucleotide binding sites are highly accommodating and translating almost every collision complex to a productive, high affinity state which may promote rapid catalysis and high turnover.

SUPPLEMENTARY TABLES

Supplementary Table 1: Results of fitting the equilibrium binding data to the sum of two Hill equations

Complex	[NaCl] (mM)	K_s(μM)	n_s	K_w (μM)	n_w	p
RecBCD·mMp	75	52 ± 8	0.99 ± 0.07	287 ± 12	12 ± 5	0.60 ± 0.04
RecBCD·mD	75	13 ± 3	0.95 ± 0.07	322 ± 7	6.2 ± 0.6	0.45 ± 0.03
RecBCD·mD	200	34 ± 5	0.8 ± 0.2	336 ± 11	8 ± 3	0.5 ± 0.10
RecBCD·mD	300	29 ± 7	1.1 ± 0.3	279 ± 11	18 ± 6	0.5 ± 0.10
RecBC·mD	75	33 ± 10	1.4 ± 0.7	300 ± 42	5 ± 2	0.4 ± 0.10
$RecB^{K29Q}C$·mD	75	23 ± 15	1.6 ± 1.2	318 ± 12	5 ± 1	0.1 ± 0.05

mD: mantADP (mD); mMp: mantAMP-pNp; K_s, K_w: Dissociation constants;
n_s, n_w: Hill coefficients, for the strong and weak phases, respectively; p: partition coefficient.

Supplementary Table 2: Number of traces in single molecule unwinding experiments

**ATP (μM)**	**20**	**100**	**200**	**350**	**500**	**1000**	**2000**
**RecBCD**	10	30	8	25	10	29	34
**RecD**	5	16	18	28	48	24	52
**RecB**	18	13	30	26	30	29	52

Supplementary Table 3: Summary of results from the transient kinetics experiments

Complex	k_{obs}^{fast}		k_{obs}^{slow}	
	K_1(μM)	k_{+2}(sec^{-1})	K_1(μM)	k_{+2}(sec^{-1})
ADP	12 ± 3	1160 ± 120	10 ± 14	134 ± 24
ADP + adenosine	26 ± 12 (μM)	1260 ± 130 (sec^{-1})		
ATP	13 ± 3	870 ± 30	235 ±16	390 ± 24
ADP + DNA	11 ± 6	1080 ± 60	11 ± 4	42 ± 5
ATP + DNA	38 ± 4	1160 ± 70	10 ± 6	125 ± 70

Supplementary Table 4: Salt-dependence unwinding rates measured by the fluorescence anisotropy assay

	High ATP		Low ATP	
**[NaCl] (mM)**	**Unwinding rate (bp sec^{-1})**	**p-value[1]**	**Unwinding rate (bp sec^{-1})**	**p-value[1]**
**75**	504 ± 13	} 4.7 $x10^{-7}$	130 ± 5	} 4.1 $x10^{-1}$
**150**	475 ± 21	} 4.9 $x10^{-4}$	128 ± 7	} 1.0 $x10^{-1}$
**200**	430± 12	} 1.2 $x10^{-11}$	123± 8	} 1.2 $x10^{-1}$
**300**	378 ± 10		118 ± 8	

[1]Two Tailed, Two sample student t-test

Supplementary Table 5: List of peptide sequences acquired from mass spectrometry analysis which contain putative auxiliary binding sites

**Putative ATP binding sites-containing peptides/amino acids**	**Amino acid position**
LLAMLEEDPTLTPR	393-406
YLPYAISDR	434-442
RWGIDDDNVRELELPATGQHTWR	503-525
L	678
QLAPLGFDLMSQKPK	688-702

Supplementary Table 6: List of all the mutations designed and the list of mutations in each mutant protein

Mutation no.	AA position and substitution
1	M393L
2	T399A
3	T401A
4	P402A
5	Y437L
6	S440A
7	I506A
8	N510G
9	L514A
10	L516A
11	T519A
12	W524L
13	R525N
14	L678A
15	L692A
16	L696A
17	M697L
18	S698A
19	K702N

Mutant	Mutation no.
$RecBC^{mut4}D$	1,6, 11, 18
$RecBC^{mut7}D$	6, 14-19
$RecBC^{mut13}D$	1-13
$RecBC^{mut19}D$	1-19

Supplementary Table 7: Steady state ATPase parameters of RecBCD WT, $RecBC^{mut7}D$ and $RecBC^{mut19}D$

**Protein**	k_{cat} **(s^{-1} RecBCD^{-1})**	$K_{M,ATP}$ **(μM)**
**RecBCD**	1134 ± 33	95 ± 16
**$RecBC^{mut7}D$**	1189 ± 36	246 ± 30
**$RecBC^{mut19}D$**	1016 ± 40	282 ± 24

Supplementary Table 8: DNA unwinding rates of RecBCD and $RecBC^{mut}Ds$ measured by the FRET assay

**Complex**	$k_{unwinding}$ (bp sec^{-1})
**RecBCD**	756 ± 35
**$RecBC^{mut4}D$**	601 ± 30
**$RecBC^{mut7}D$**	630 ± 31
**$RecBC^{mut13}D$**	205 ± 10
**$RecBC^{mut19}D$**	187 ± 16

Supplementary Table 9: Equilibrium constants for nucleotides binding to RecBCD and $RecBC^{mut}Ds$

**Complex**	**[NaCl] (mM)**	K_D **(μM)**	**$\Delta G^{\circ\prime}_D$ (kJ mol^{-1})**
**RecBCD·mADP (strong phase)**	75	13 ± 3	-27.8 ± 0.6
**$RecBC^{mut4}D$·mADP**	75	83 ± 16	-23.3 ± 1.0
**$RecBC^{mut7}D$·mADP**	75	114 ±17	-22.5 ± 1.1
**$RecBC^{mut13}D$·mADP**	75	148 ± 54	-21.8 ± 1.9
**$RecBC^{mut19}D$·mADP**	75	238 ± 61	-20.7 ± 2.1
**$RecBC^{mut19}D$·mADP + adenosine**	75	195± 20	-21.2 ± 1.1

1. mantADP (mADP)
2. Fitted parameters for the hyperbolic equation, K_D= Equilibrium dissociation constants for the strong phase
3. $\Delta G^{\circ\prime}_D$(kJ mol^{-1}) = -RTln(K_D) is the standard binding energy at 1 M ligand, R is the gas constant (8.314J $mol^{-1}K^{-1}$), T is the absolute temperature in Kelvin.

Supplementary Table 10: Summary of results from the global fit to the model

Kinetic Parameter	Value	
$k_s^+ (\mu M^{-1}\ \text{sec}^{-1})$	56 ± 3	
$k_s^- (\text{sec}^{-1})$	678 ± 1	
$k_w^+ (\mu M^{-1}\ \text{sec}^{-1})$	$\alpha\ (\mu M^{-1}\ \text{sec}^{-1}\ mM^{-1})$	-4 ± 1
	$\beta\ (\mu M^{-1}\ \text{sec}^{-1})$	10 ± 2
$k_w^- (\text{sec}^{-1})$	$\gamma\ (\text{sec}^{-1}\ mM^{-1})$	-53 ± 3
	$\delta\ (\text{sec}^{-1})$	36,500 ± 29
$k_{tr}^+ (\text{sec}^{-1})$	$\epsilon\ (\text{sec}^{-1}\ mM^{-1})$	-487 ± 2
	$\kappa\ (\text{sec}^{-1})$	178,000 ± 1,600
$k_{tr}^- (\text{sec}^{-1})$	35,400 ± 1,100	
a	19 ± 1	
$v_{max} (\text{bp sec}^{-1})$	1,250 ± 4	

Supplementary Table 11: Oligonucleotides used for building DNA substrates for Optical Tweezers experiments

**Construct Name**	**Oligonucleotide sequence (5'→3')**
Biotin 600 bp track	/5'Biotin/-GCTTTAATGCGGTAGTTTATCA
	GCAGCATTAGGAAGCAGCCCAGGCATTAGGAAG CAGCCCAG
Dig 600 bp track	/5' Phosphate/- GCATTAGGAAGCAGCCCAGGCTTTATTGCGGTAG TTTATCA
	/5'digoxigenin/-GCATTAGGAAGCAGCCCAG
Biotin 4000 bp track	/5'Biotin/-GCTTTAATGCGGTAGTTTATCA
	GCACTACGCCTCAGCTTGCCCCTCAGCGATGACCTCAG CATTCCCTTTTTTGCGGCATT
Dig 4000 bp track	/5'Phosphate/- CTACGCCTCAGCTTGCCCCTCAGCGATGACCTCAGCGT CACTGGTCCCG
	/5'digoxigenin/-GCATTAGGAAGCAGCCCAG
Biotin short track	/5'Biotin/AACCACCAACCAACAACCACCCAAACCCAAA CCCAAGGTCATCGCTGAGGGGC AAGCTGAGGCGTAGTGC
Dig short track	/5'Phasphate/CTACGCCTCAGCTTGCCCCTCAGCGATGAC CAAACCACCAACCAACAACCACCCAAACCCAAACCCA CAC/3'digoxigenin /
Stem, forward	ATGGCCTTGCCGGCCGATGGACCCTATACGCGGCC
Stem, reverse	ATCACTGCGTGGAATTCGATATCCCCGAGAAGG

Supplementary Table 12: Sequences of the DNA substrates used for DNA unwinding experiments

#	Sequence description	Sequence 5' – 3'	nts
1	Anisotropy 24 bp	GGAAGAGAGGAAGAGAGGAAGAGAGGAAGAG GGAGG GAGGGAGGGTTTTCCCTCCCTCCCTCCCTCTTCC	70
2	Anisotropy 24 bp FAM	FAM-TCTCTTCCTCTCTTCCTCTCTTCC	24
3	Anisotropy 38 bp	AAGAGAGGAAGAGGAAGAGAGGAAGAGAGGA AGAGAGGA AGAGGGAGGGATTTTTCCCTCCCTCTT	66
4	Anisotropy 38 bp FAM	FAM- CCTCTCTTCCTCTCTTCCTCTCTTCCTCTTCCTCT CTT	38
5	Anisotropy 52 bp	GAGAGGAAGAGAGGAAGAGAGGAAGAGGAAG AGAGGAAG AGAGGAAGAGAGGAAGAGGGAGGGATTTTTCC CTCCCTCT T	80
6	Anisotropy 52 bp FAM	FAM - CCTCTCTTCCTCTCTTCCTCTCTTCCTCTTCCTCT CTTCCTC TCTTCCTCTC	52
7	Anisotropy 62 bp	GAGGAGAGGAAGAGAGGAAGAGAGGAAGAGG AAGAGAGGAAGAGAGGAAGAGAGGAAGAGGG CGGGATTTTTATCCCG	78
8	Anisotropy 62 bp FAM	FAM- CCCTCTTCCTCTCTTCCTCTCTTCCTCTCTTCCTC TTCCTCTCTTCCTCTCTTCCTCTCCTC	62
9	hpDNA	CATGTGACTCGTTACCTGAGTTTTTACTCAGGTA ACGAGT CACATG	46
10	FRET 62 bp 5Cy3	5Cy3- CCCTCTTCCTCTCTTCCTCTCTTCCTCTCTTCCTC TTCCTCTCTTCCTCTCTTCCTCTCCTC	62
11	FRET 62 bp 3Cy5	GAGGAGAGGAAGAGAGGAAGAGAGGAAGAGG AAGAGAGGAAGAGAGGAAGAGAGGAAGAGGG- 3Cy5	62

FAM: 6-fluorescein amidi

SUPPLEMENTARY FIGS

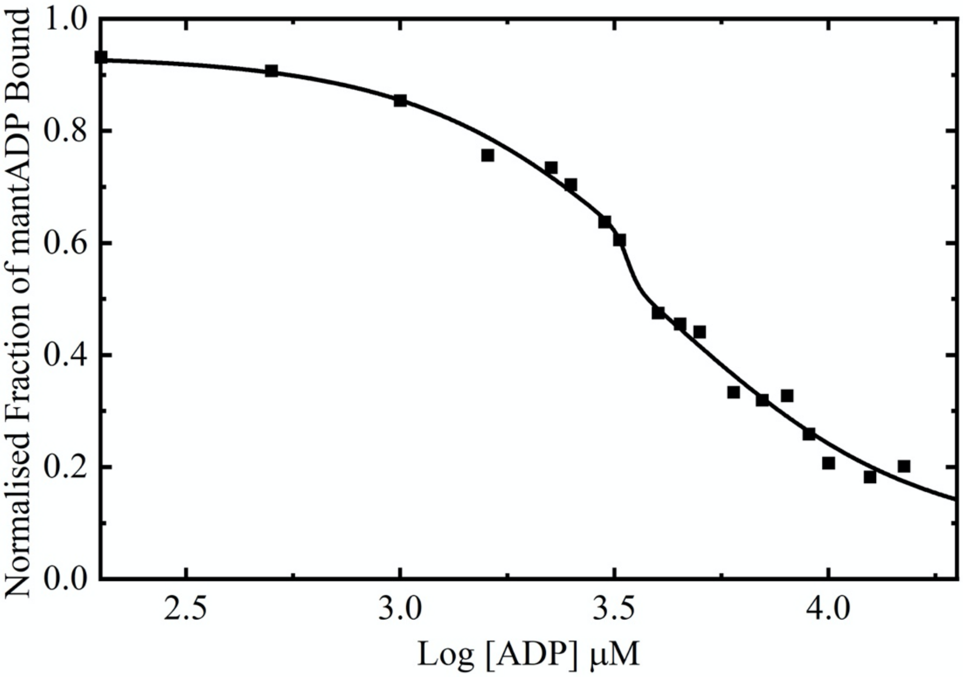

Supplementary Fig. 1: Equilibrium binding competition of unmodified nucleotides to RecBCD·mantADP complexes. Pre-bound mantADP (600 μM) was competed with titrated unlabeled ADP. MantADP dissociation curve displays a biphasic pattern: both nucleotide-binding components, the lower and the higher affinity regimes are well competed off with the unlabeled ADP. The data were fit by using the log of the weighted sum of two hyperbolas. n = 1 experiment.

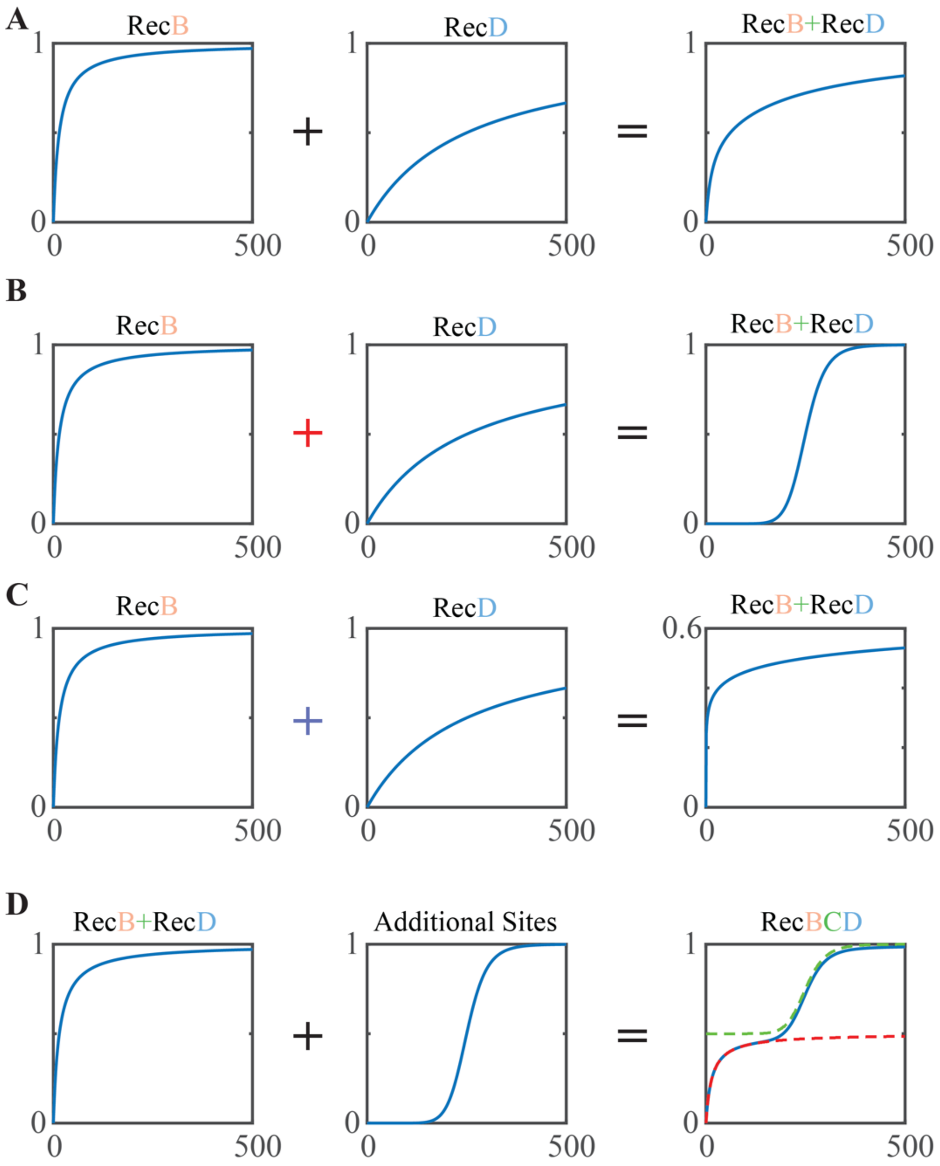

Supplementary Fig. 2: Models for equilibrium nucleotide binding to RecBCD. A. Nucleotide binding to RecBCD as a result of two binding sites with different affinities. **B.** Nucleotide binding to RecBCD as a result of binding among two (catalytic) sites with positive cooperativity (red plus sign). **C.** Nucleotide binding to RecBCD as a result of binding among two sites (catalytic) with negative cooperativity (blue plus sign). **D.** Nucleotide binding to RecBCD in the presence of non-cooperative binding to the catalytic sites and cooperative non-catalytic sites with lower affinity. The decomposition for the catalytic (red) and additional (green) sites is shown in dashed lines.

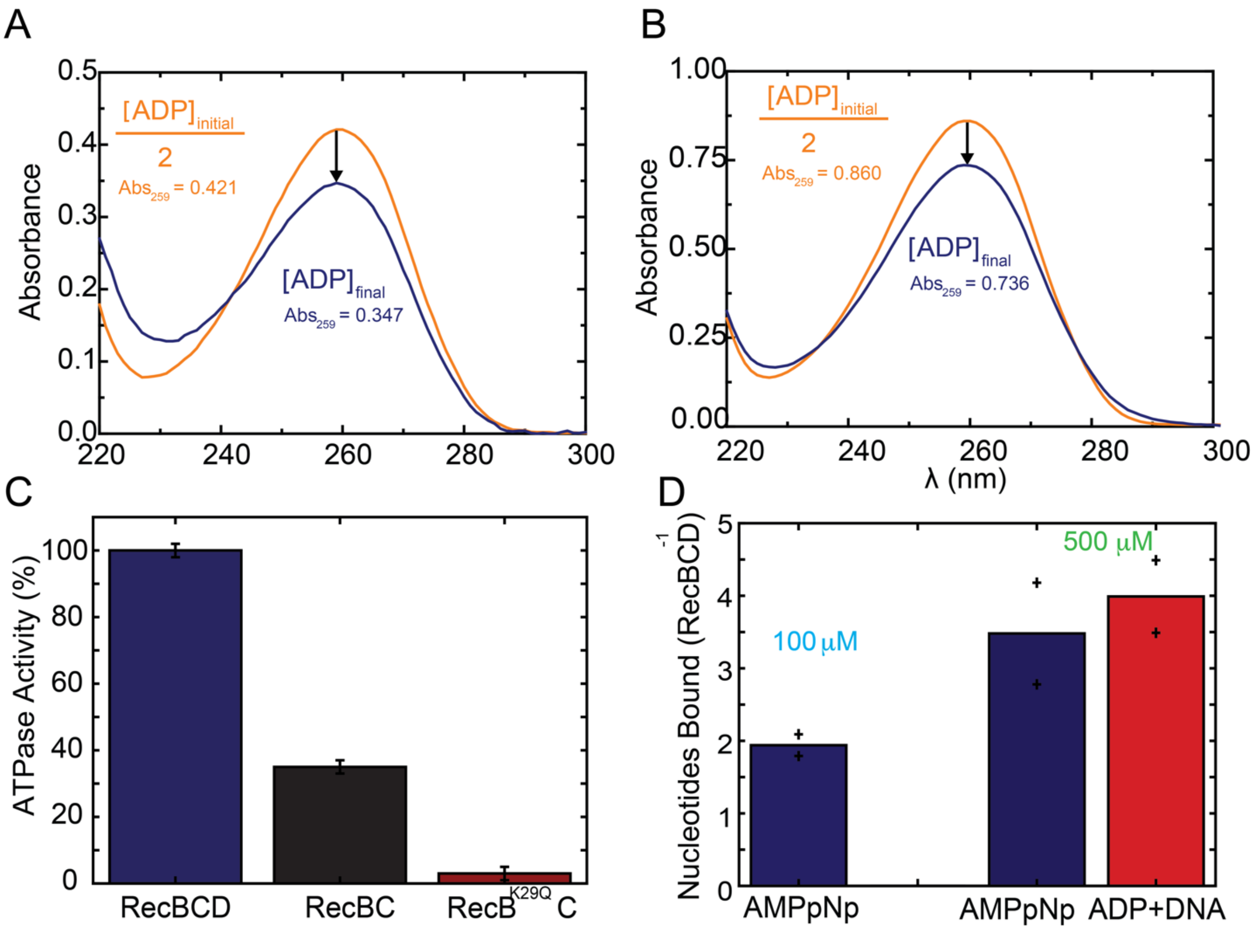

Supplementary Fig. 3: Equilibrium dialysis experiments. A. Absorbance spectra of 4x diluted ADP pre- (orange, divided by a factor of two) and post- (blue) equilibrium dialysis with 17 μM RecBCD. The net change in the absorption indicates ~2 nucleotide binding sites per RecBCD. Data shown as mean, n = 2. **B.** Absorbance spectra of 10x diluted ADP pre- (orange, divided by factor of two) and post- (blue) equilibrium dialysis with 45 μM RecBCD. The net change in the absorption indicates ~4 nucleotide binding sites per RecBCD. Data shown as mean, n = 4. **C.** Relative ATPase activity of RecBCD, RecBC and the catalytically deficient mutant $RecB^{K29Q}CD$, at saturating ATP (2 mM) and *E.coli* DNA (300 nM), obtained from the steady state initial rates (error bars are the s.e. of the fit). **D.** Number of nucleotides (blue: AMPpNp, green: ADP) bound to RecBCD (blue) and pre-incubated RecBCD·DNA (green), measured and analyzed as in Fig. 1. n = 2 independent experiments.

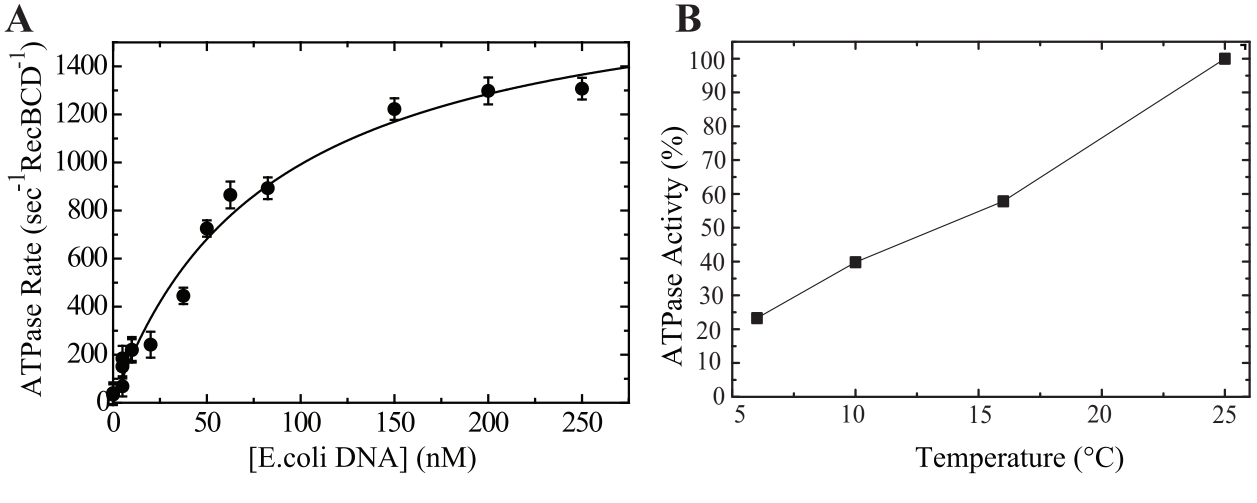

Supplementary Fig. 4: Steady-state ATPase activity of RecBCD with *E.coli* DNA. A. *E.coli* DNA concentration-dependence of RecBCD steady state ATP turnover velocity (ATPase activity). The solid lines through the data points are the best fits to a quadratic form of the Briggs-Haldane equation (Eq. 2, Methods). The steady state parameters are K_M = 90.9 ± 18.3 nM and k_{cat} = 1,862 ± 143.1 s^{-1} $RecBCD^{-1}$. [*E.coli*-DNA] refers to the concentration of blunt ends in digested *E.coli* chromosomal DNA. Data shown as mean ± s.e.m., n=3. **B.** Temperature dependence of RecBCD ATPase activity at saturating ATP (2 mM) and *E.coli* DNA (300 nM), obtained from the steady state initial rates (error bars are the s.e. of the fit), and shown as a percent of the mean activity at 25º C.

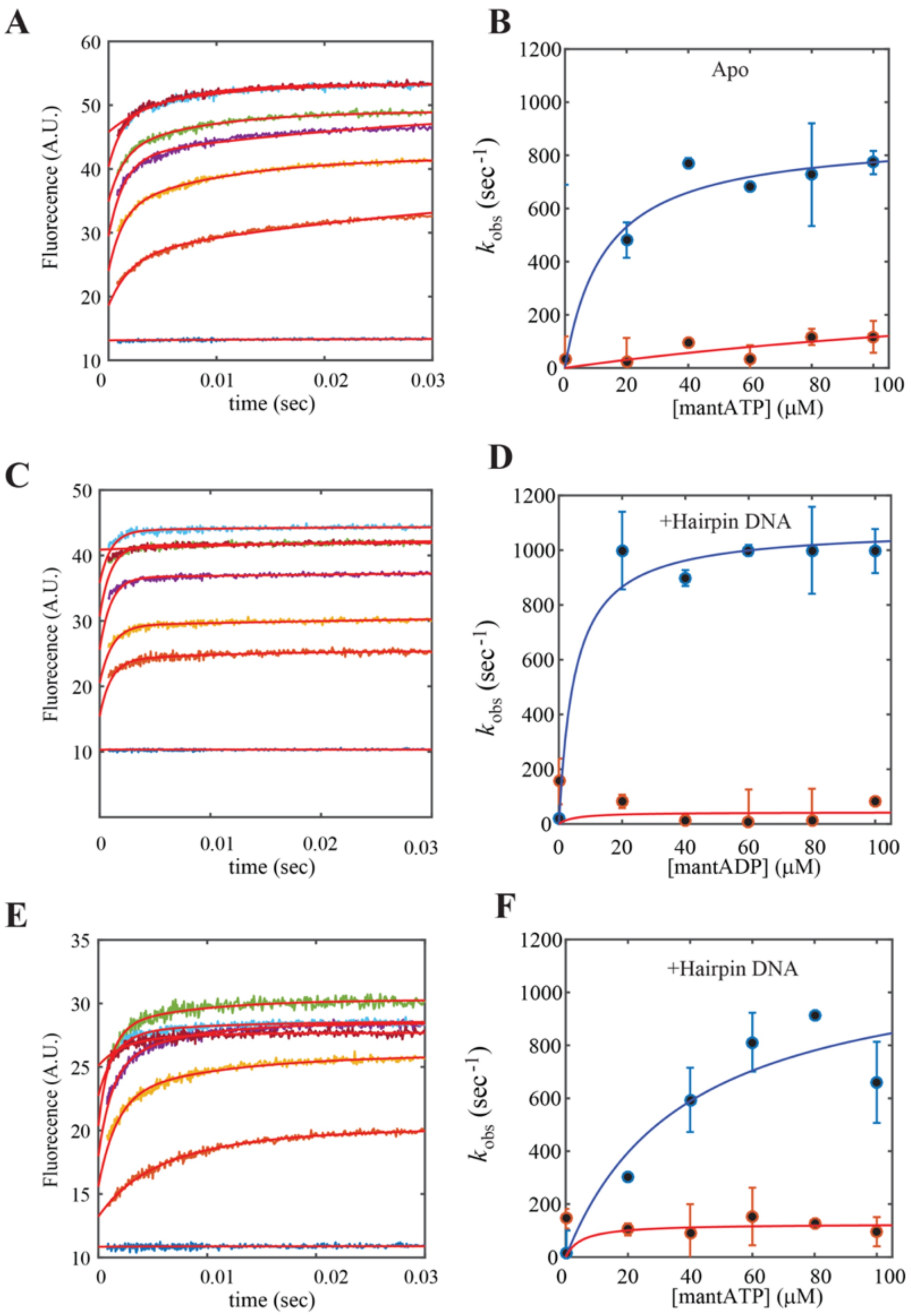
A
Fluorecence (A.U.)
time (sec)
B
Apo
k_{obs} (sec^{-1})
[mantATP] (μM)
C
Fluorecence (A.U.)
time (sec)
D
+Hairpin DNA
k_{obs} (sec^{-1})
[mantADP] (μM)
E
Fluorecence (A.U.)
time (sec)
F
+Hairpin DNA
k_{obs} (sec^{-1})
[mantATP] (μM)

Supplementary Fig. 5: Transient kinetics of mant-nucleotides binding to RecBCD in the absence and presence of DNA. A. Time courses of mant-ATP binding upon rapid mixing of RecBCD (2 µM, post-mixing) with mantATP (0-100 µM, lower to upper, respectively). The red lines through the data are the best global fit to a double exponential function (Methods). **B.** Dependence of k_{obs}^{fast} (Blue) and k_{obs}^{slow} (Orange) on [mantATP], as obtained from fitting n = 4-5 independent measurements for each concentration. The error bars represent the standard error obtained from the fit. The solid line through the data points are best hyperbolic fits. **C.** Time courses of mantADP binding upon rapid mixing of RecBCD·HairpinDNA (2 µM, 1:1.1 ratio, post-mixing) with mantADP (0-100 µM, lower to upper, respectively). The red lines through the data are the best global fit to a double exponential function (Methods). **D.** Dependence of k_{obs}^{fast} (Blue) and k_{obs}^{slow} (Orange) on [mantADP], as obtained from fitting n = 4-5 independent measurements for each concentration. The error bars represent the standard error obtained from the fit. The solid line through the data points are best hyperbolic fits. **E.** Time courses of mantATP binding upon rapid mixing of RecBCD·HairpinDNA (2 µM,1:1.1 ratio, post-mixing) with mantATP (0-100 µM, lower to upper, respectively). The red lines through the data are the best global fit to a double exponential function (Methods). **F.** Dependence of k_{obs}^{fast} (Blue) and k_{obs}^{slow} (Orange) on [mantATP], as obtained from fitting n = 4-5 independent measurements for each concentration. The error bars represent the standard error obtained from the fit. The solid line through the data points are best hyperbolic fits.

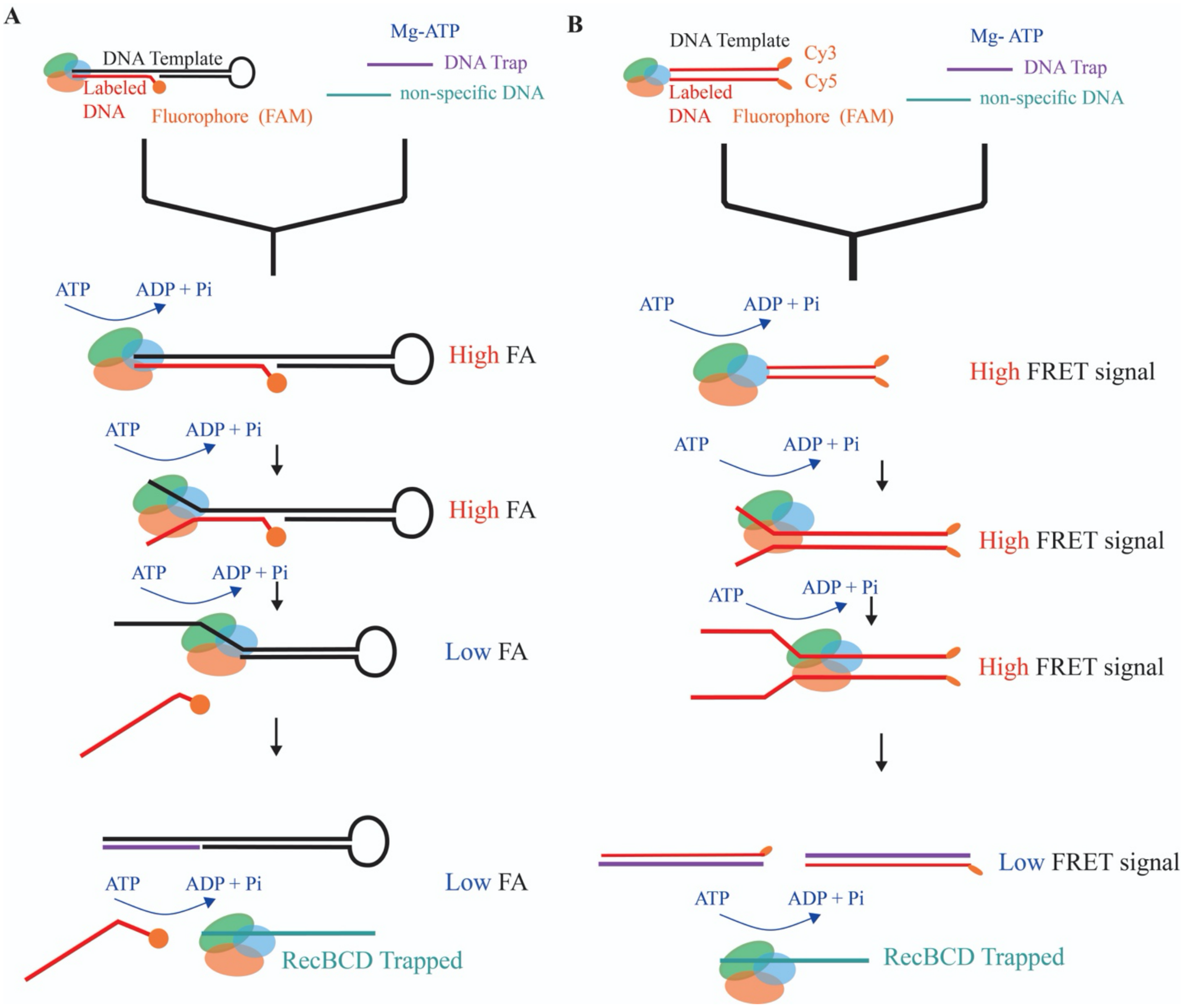

Supplementary Fig. 6: Rapid mixing assays to measure real time unwinding by RecBCD. A. Schematic description of the Fluorescence Anisotropy (FA) assay. A pre-bound RecBCD/labeled-DNA complex is mixed with ATP and DNA traps designed to capture the released RecBCD and DNA template. A high FA phase corresponds to the unwinding phase, while a lower FA corresponds to the dissociation of RecBCD from the DNA template. **B.** Schematic description of the FRET assay. A pre-bound RecBCD/labelled-DNA complex is mixed with ATP and DNA traps designed to capture the released RecBCD and DNA template. A pre-bound RecBCD/labelled-DNA complex displays high FRET signal as long as there is unwinding. After RecBCD unwinds the DNA substrate and dissociates from it, the FRET signal is low**.**

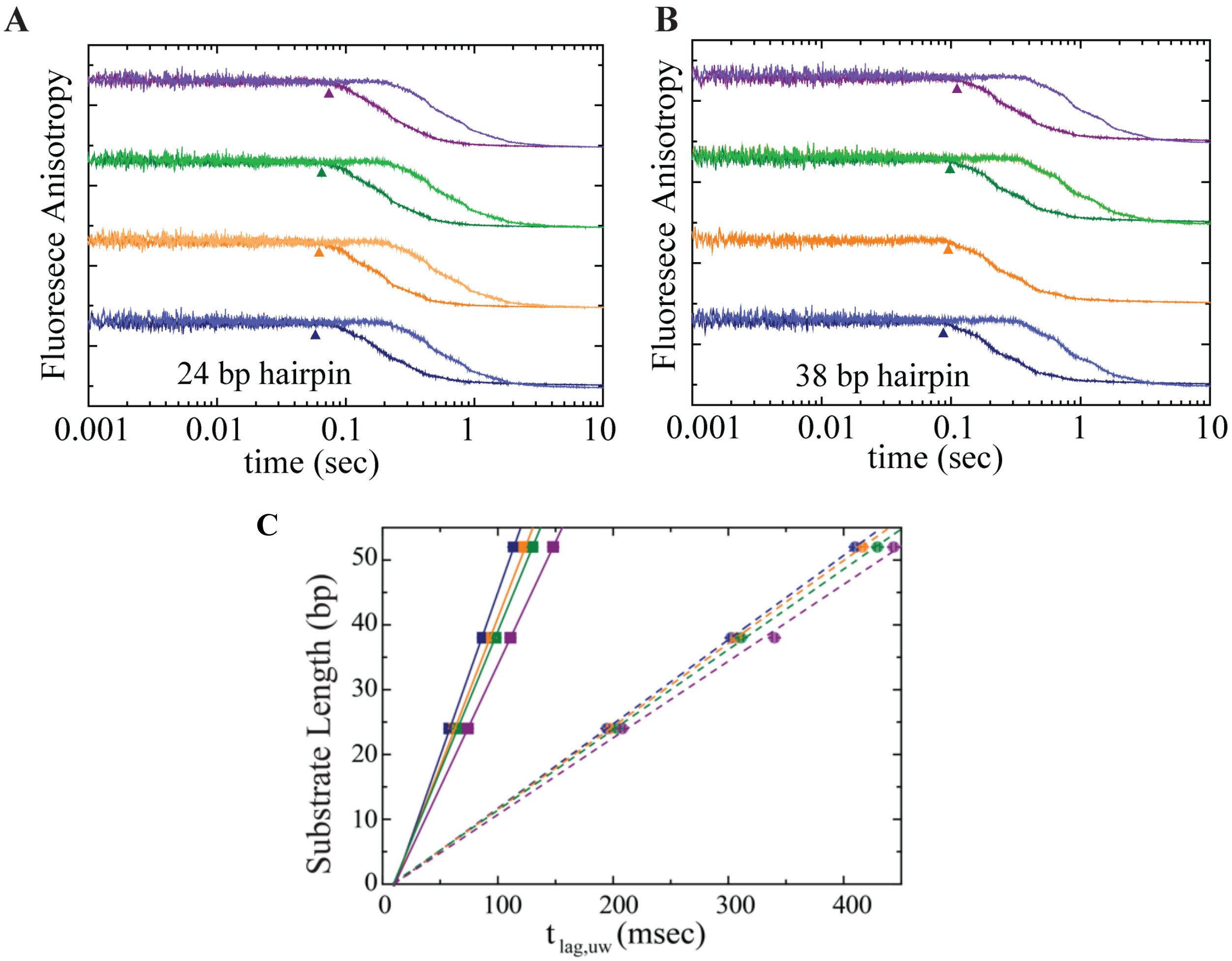

Supplementary Fig. 7: Salt dependence of the unwinding rates. A. and **B.** FA time courses of unwinding reactions of a post-mixed 250 nM RecBCD·hpDNA (24bp **A,** and 38bp **B**) with [ATP] = 350 µM (strong colors) and [ATP] = 100 µM (light colors). Difference colors indicate time traces at 75 mM (blue), 150 mM (orange), 200 mM (green), and 300 mM (purple) NaCl concentrations. **C.** Unwinding lag duration as a function of the substrate length at high (350 µM, squares) and low (100 µM, circles) ATP concentrations. Data shown as mean ± s.e.m., n = 11 independent measurements for each point. Lines through the data are best linear fits.

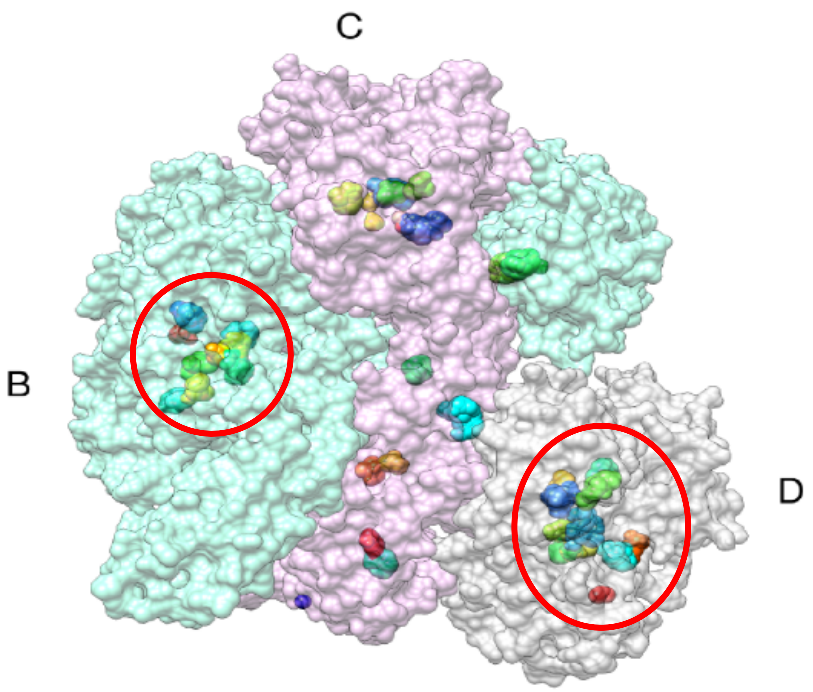

Supplementary Fig. 8: FTMap computation of RecBCD (PDB 1w36). RecBCD is presented in a space filling model, with chains colored turquoise (RecB), pink (RecC) and gray (RecD). Bound FTmap probes are shown in different colors. The canonical ATP binding sites (Walker A and B) are circled in red.

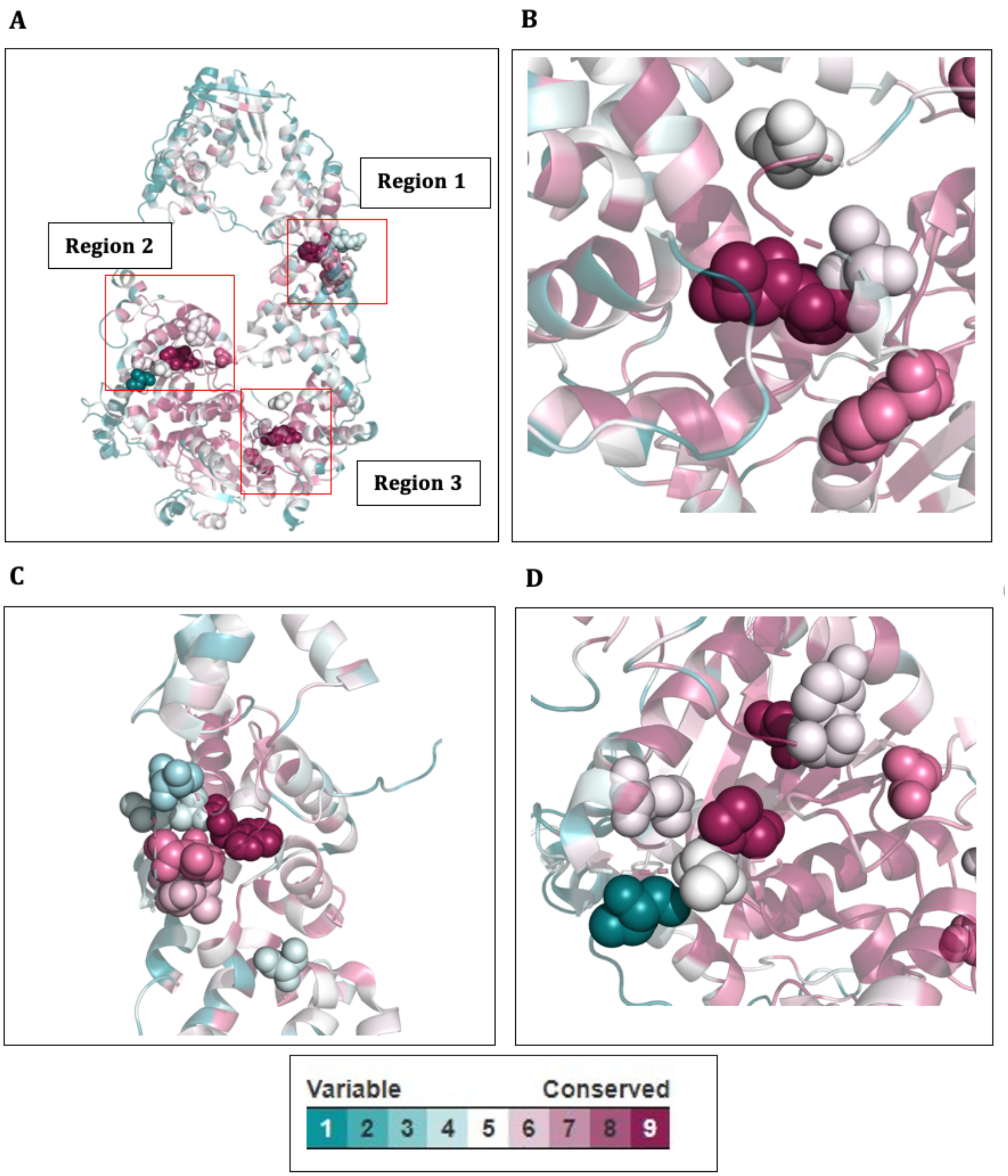

Supplementary Fig. 9: Conservation of mutated residues A. RecC subunit showing the three major regions with mutated peptide sequences. Zoomed in view of **B.** Region 1 **C.** Region 2 and **D.** Region 3. The mutated amino acids are shown as spheres to distinguish them from the overall cartoon representation, and colored according to their conservation score.

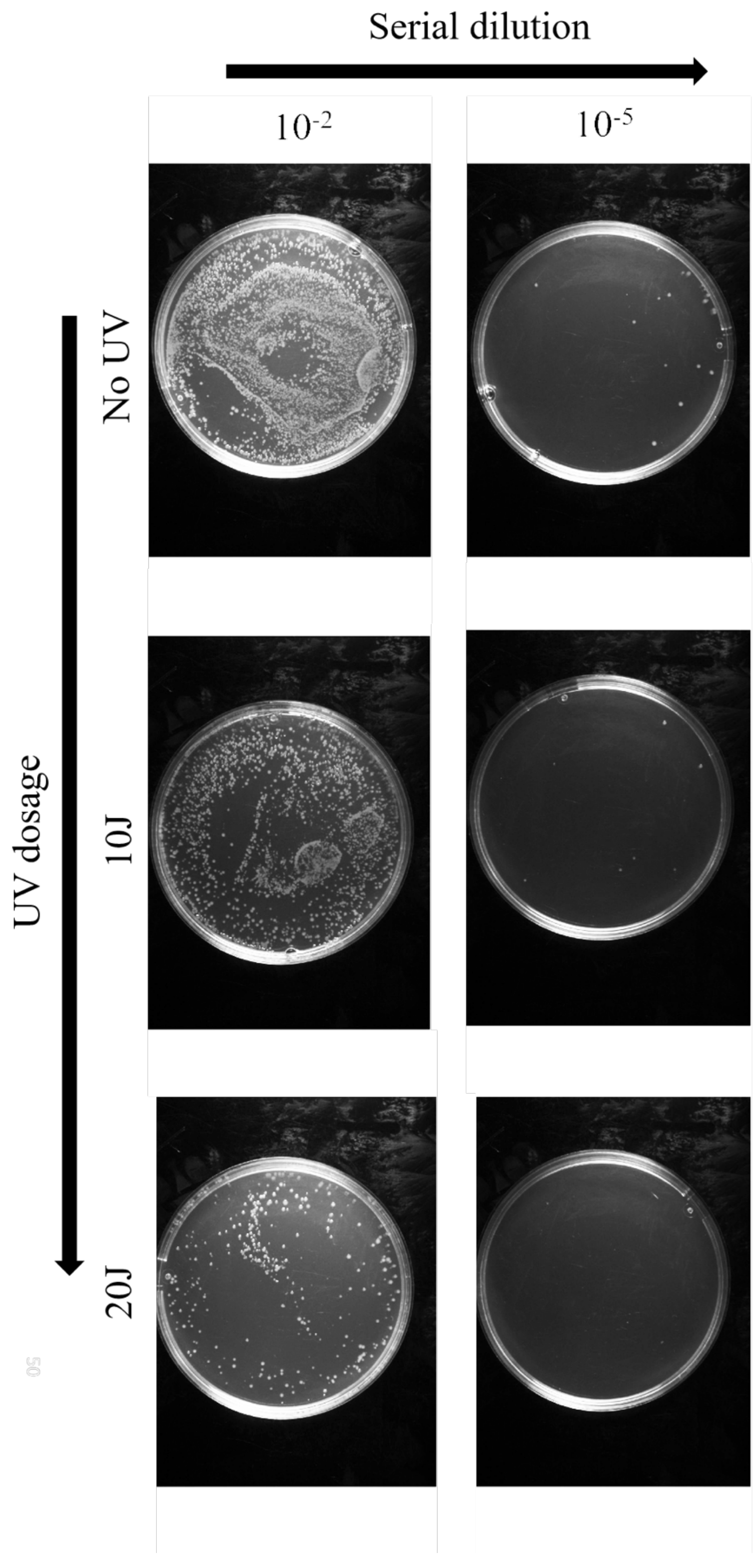

Supplementary Fig. 10: Agar plates for survival assay. Representative Agar plates demonstrating loading a 100 µl bacterial sample (in this example, WT) for each UV dose and two serial dilutions.

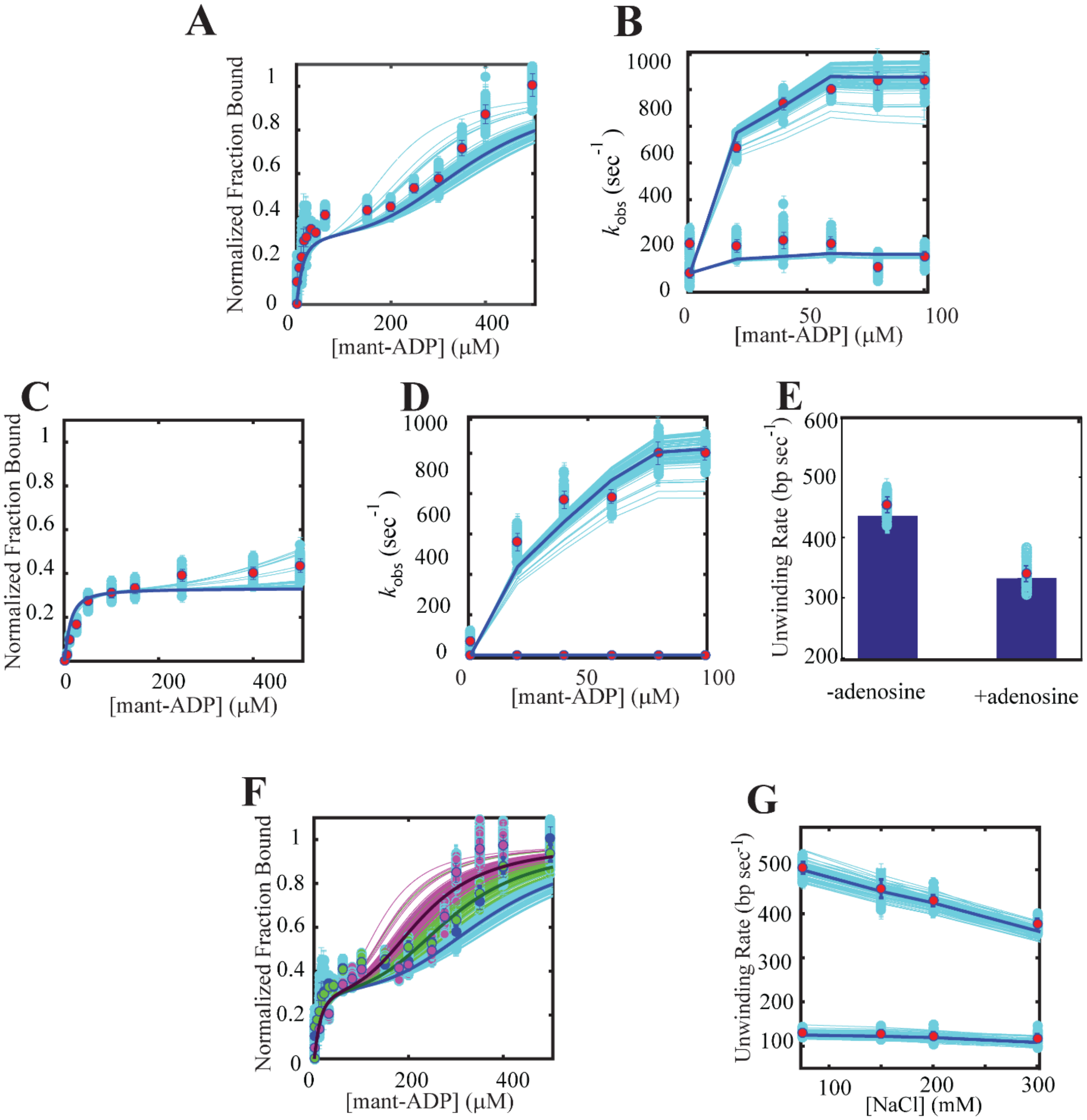

Supplementary Fig. 11: Global fitting of the kinetic scheme to the measured binding and unwinding data. A-G. Data is shown in circles and results of the model in full lines. The light-blue lines show the results of n = 100 bootstrap Monte Carlo runs (see Methods). **A.** Equilibrium binding of mantADP at normal conditions (75 mM NaCl). Data shown as in Figure 1A. **B.** Transient kinetics of mantADP binding at normal conditions. Data shown as in Figure 3B. **C.** Equilibrium binding of mantADP in the presence of 2 mM adenosine. Data shown as in Figure 1B. **D.** Binding kinetics of mant-ADP in the presence of 2 mM adenosine. Data shown as in Figure 3D. **E.** Unwinding rates in the presence of 2 mM adenosine. Data shown as in Figure 4D. **F.** Equilibrium binding of mantADP at varying salt concentrations (75, 200, 300 mM NaCl in blue green and purple, respectively). Data shown as in Figure 1B. **G.** Unwinding rates as a function of salt for low (lower) and high ATP (higher). Data shown as in Figure 4B.

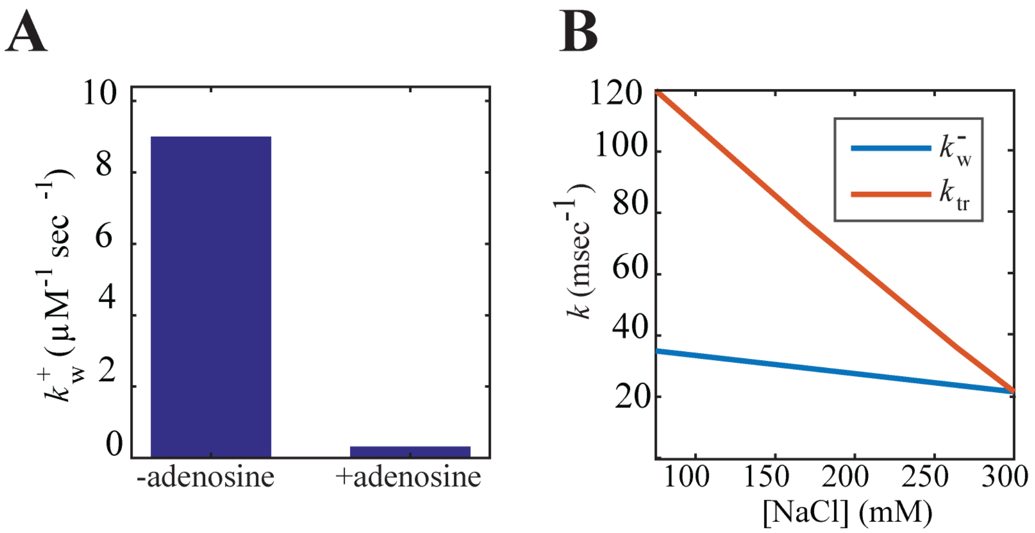

Supplementary Fig. 12: Model parameters as a function of adenosine and salt. A. k_w^+ in the presence and the absence of 2 mM adenosine. **B.** k_w^-, k_{tr}^+ as a function of [NaCl].

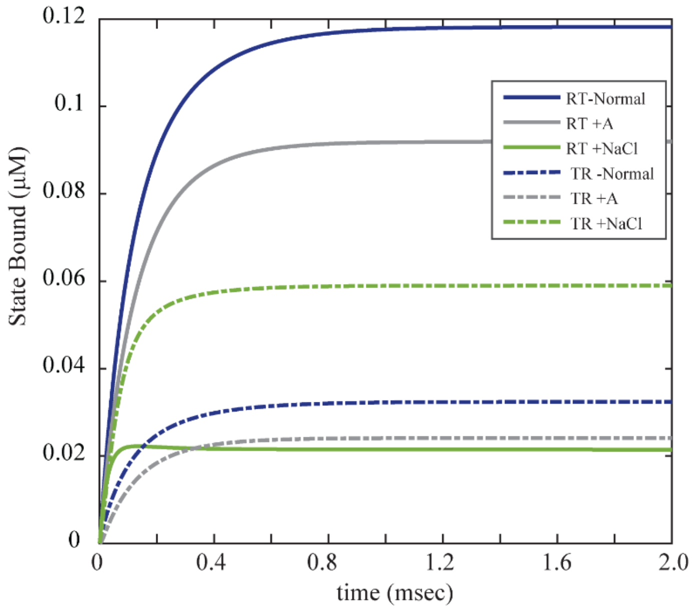

Supplementary Fig. 13: Modeled rapid mixing shows the effect of adenosine and salt on the rates and occupancies of the catalytic sites. Rapid mixing modeling of 40 µM ATP with 1 µM RecBCD in different buffer conditions at 25º C (blue, 75 mM NaCl, gray 75 mM NaCl in the presence of 2 mM adenosine, and green for 300 mM NaCl).

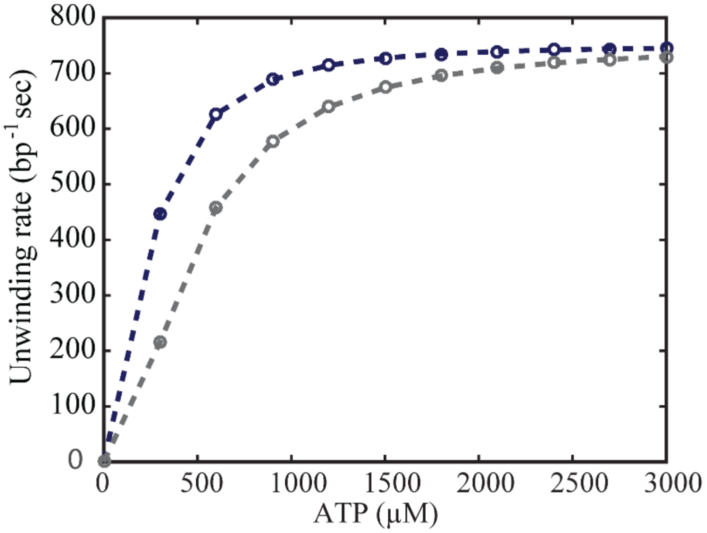

Supplementary Fig. 14: ATP-dependent unwinding rates indicate a hyperbolic-like pattern. Simulations of ATP-dependent unwinding rate by RecBCD, in the absence (blue) and in the presence (grey) of 2 mM adenosine, indicate hyperbolic-like curves that resemble Michaelis-Menten behavior.

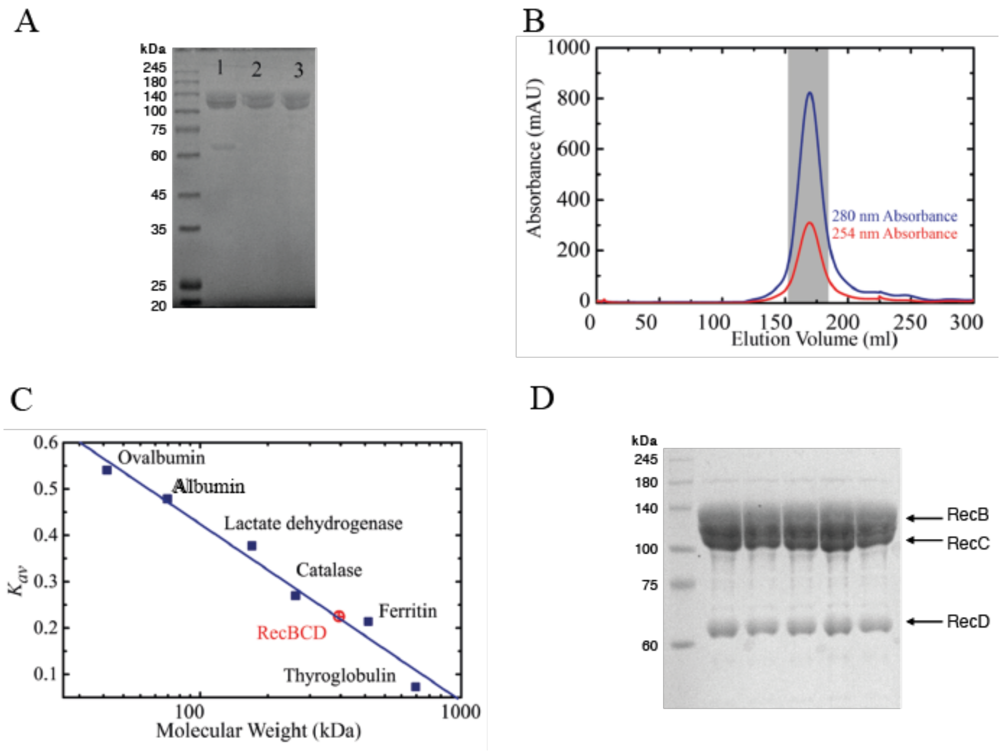

Supplementary Fig. 15: Purification and analysis of RecBCD oligomeric state. A. Coomassie stained 10% SDS-PAGE of RecBCD and constructs purifications. The left lane is the MW standards and their corresponding MW in kDa. Followed by RecBCD **(**lane 1**),** RecBC (lane 2) and RecB^{K29Q}C (lane 3). The experiment was repeated two times independently with similar results. **B.** Size Exclusion Chromatography (SEC) analysis of RecBCD purification showing a major mono disperse peak. The major peak, which correspond to RecBCD, was collected during fractionation (highlighted in grey). **C.** Determination of RecBCD MW was performed by using MW standards proteins (blue squares) using analytical SEC. The solid line shows a linear fit of the *K*av as a function of the log MW (kDa). RecBCD predicted MW according to the known standards K_{av} values is calculated to be ~340 kDa (shown as red circle). MW standards are: Ovalbumin (43 kDa), Albumin (66 kDa), Lactate dehydrogenase (140 kDa), Catalase (232 kDa), Ferritin (440 kDa), Thyroglobulin (669 kDa). **D.** Coomassie stained 10% SDS-PAGE of RecBCD **(**lane 1**),** RecBC^{mut4}D (lane 2), RecBC^{mut7}D (lane 3), RecBC^{mut13}D (lane 4) and RecBC^{mut19}D (lane 5) purifications. RecC^{mut}, RecB and RecD are indicated with arrows. The left lane is the MW standards and their corresponding MW in kDa. Two experiment was repeated two times independently with similar results.

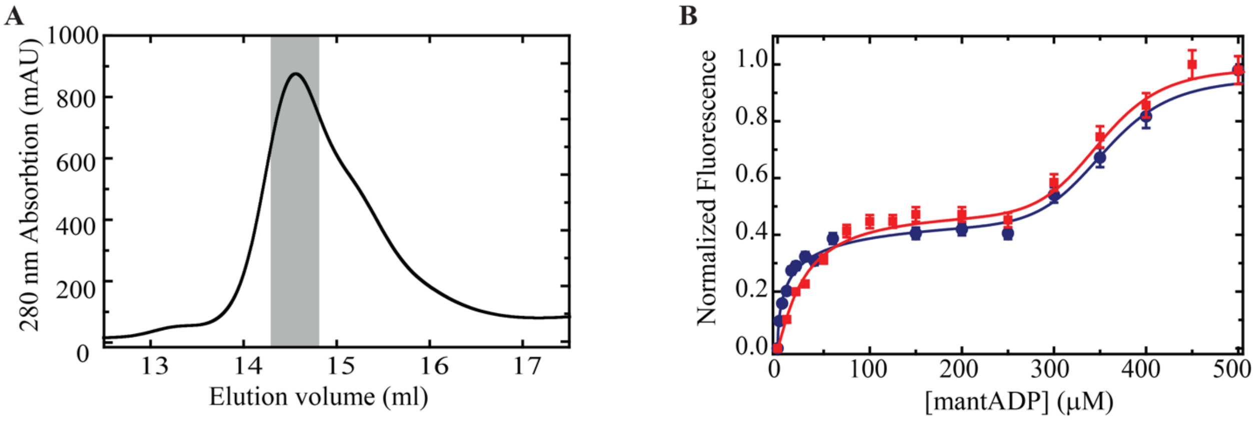

Supplementary Fig. 16: MonoQ-purified RecBCD exhibits a biphasic binding pattern. A. RecBCD was purified following the same protocol as described in the 'Online Materials', immediately subjected to an additional MonoQ purification step and eluted with a salt gradient. The shaded area shows the fraction collected. **B.** The MonoQ-purified protein was tested for nucleotide binding and compared with the titration in Fig. 1B, showing nearly identical biphasic binding. Lines show the best fit to Eq. 1 (Methods).

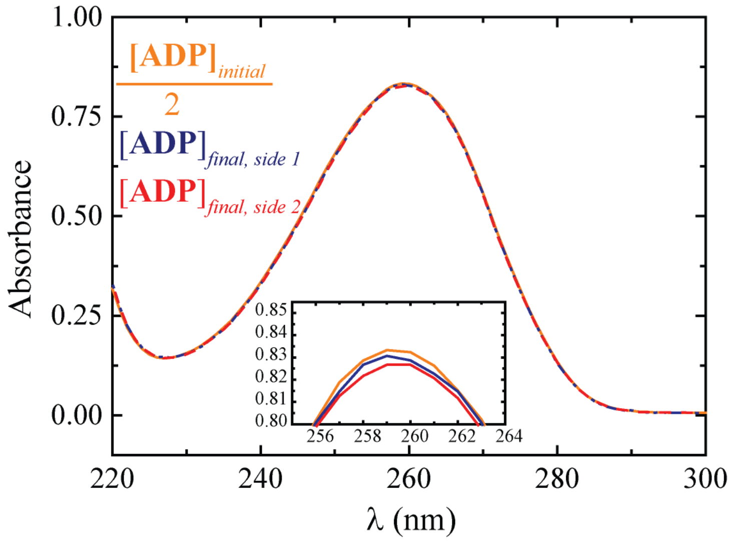

Supplementary Fig. 17: Calibration of the equilibrium dialysis experiments indicates minimal loss across the membrane. Absorbance spectra of ADP: 20x diluted, before (yellow) and 10x diluted, after (dashed blue, side 1 and dashed red, side 2) equilibrium dialysis. The initial half absorbance at 259 nm was determined as 0.8332 (corresponding to 0.5411 M of ADP), the post-dialysis absorbance was determined as 0.8307 (0.5394 M of ADP) on the left side and 0.8267 (0.5368 M ADP) on the right side, indicating a total loss of 0.006 M of ADP and a 0.54% error. **Inset:** Zoom-in to the peaks at 259 nm.

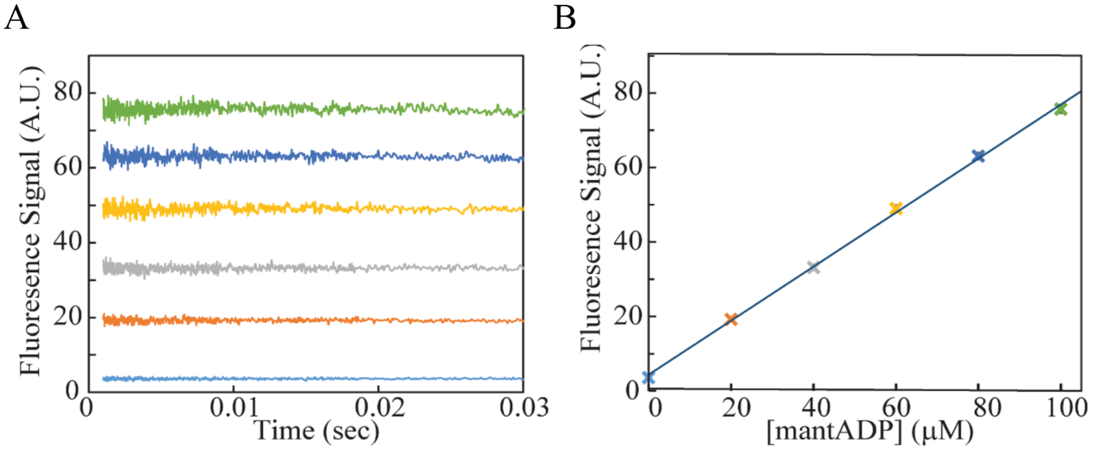

Supplementary Fig. 18: Fluorescence background quantification for binding kinetics experiments. A. Time courses of fluorescence upon rapid mixing of buffer with mantADP (mantADP 0-100 µM, lower to upper, respectively). **B.** The time-averaged fluorescence signal displays a linear dependence on mantADP concentrations.

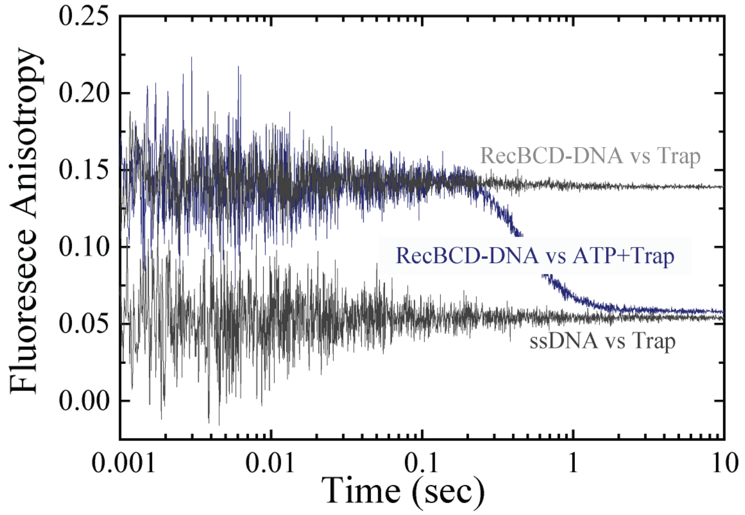

Supplementary Fig. 19: Fluorescence Anisotropy time courses reflect the physical state of RecBCD·DNA interaction. Anisotropy time courses of rapidly mixing RecBCD·hpDNA (38bp) vs. buffer containing traps (light grey), ssDNA vs. a buffer containing traps (dark grey), and RecBCD·hpDNA vs. ATP. In the presence of ATP, the anisotropy signal drops from the value of RecBCD-bound DNA to ssDNA free in solution with a time lag representing the time taken for unwinding.

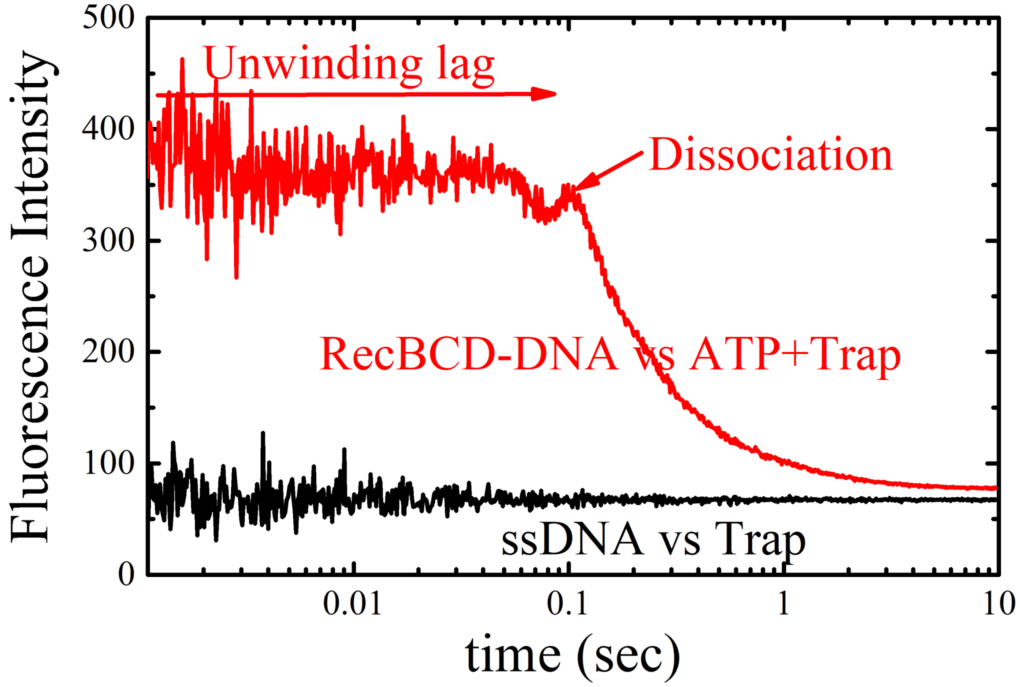

Supplementary Fig. 20: FRET time courses reflect the physical state of RecBCD·DNA interaction. FRET time courses of rapidly mixing ssDNA vs. a buffer containing traps (black) and RecBCD·hpDNA vs. ATP and trap (red). In the presence of ATP, high FRET signal phase corresponds to the unwinding phase and the FRET signal drops to ssDNA free in solution with a time lag representing the time taken for unwinding.

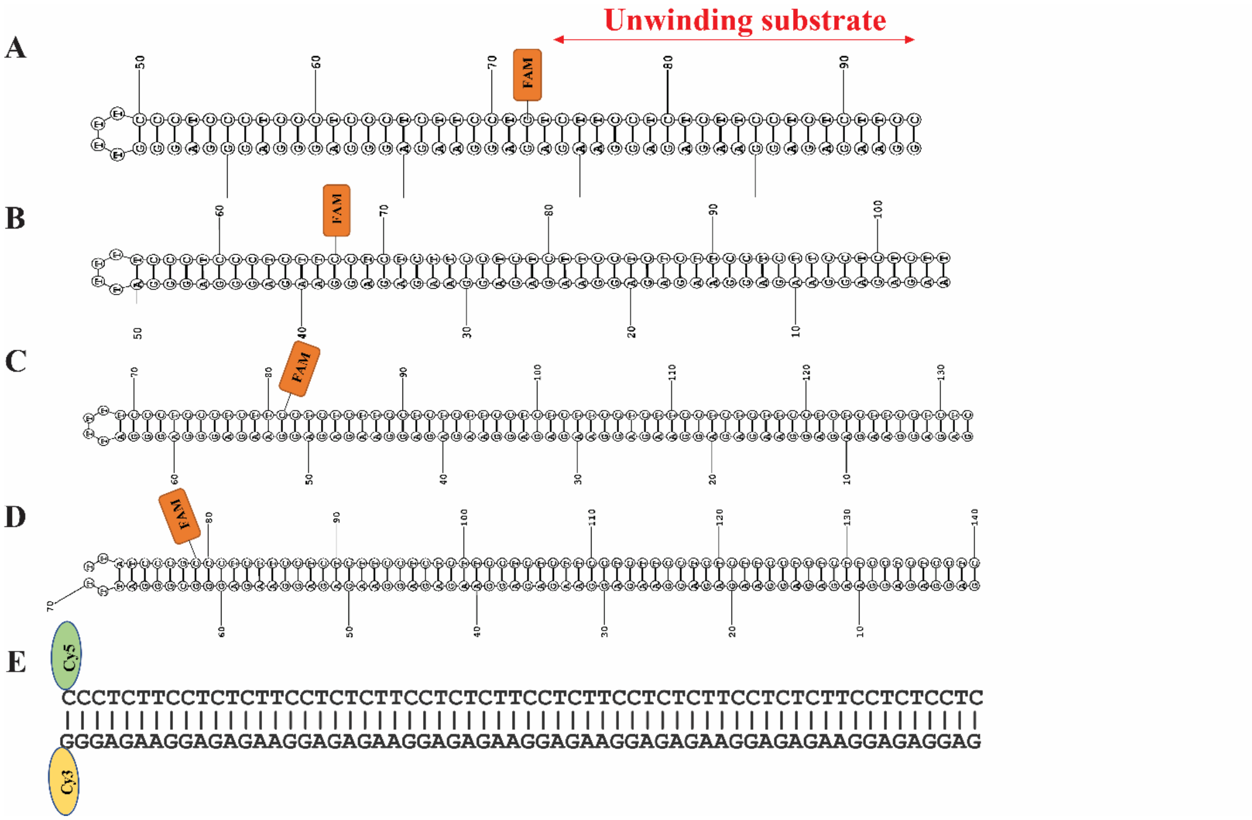

Supplementary Fig. 21: **Schematic representation of DNA substrates used for the ensemble unwinding experiments. A.** 24bp, **B.** 38bp, **C.** 52bp, **D.** 62bp and **E.** 62bp Cy3-Cy5 used for FRET unwinding assay

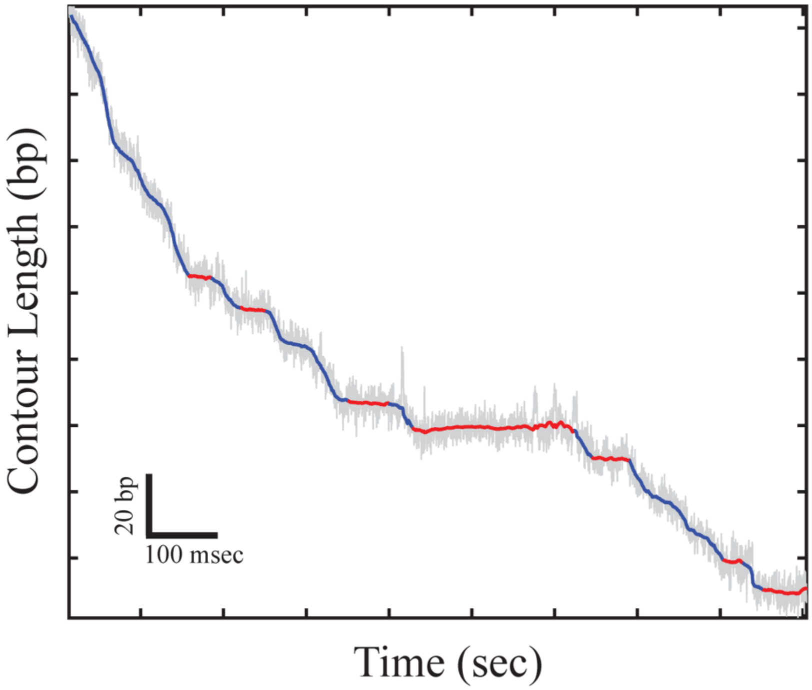

Supplementary Fig. 22: Chung-Kennedy filter and pause detection analysis of single-molecule experiments. Representative trace (ATP 2 mM, AMPpNp 300 μM). Raw data is shown in gray. Data filtered with a Chug-Kennedy filter (Methods) is represented in blue and red; where blue represents translocation phases and red represents pauses detected by our algorithm.

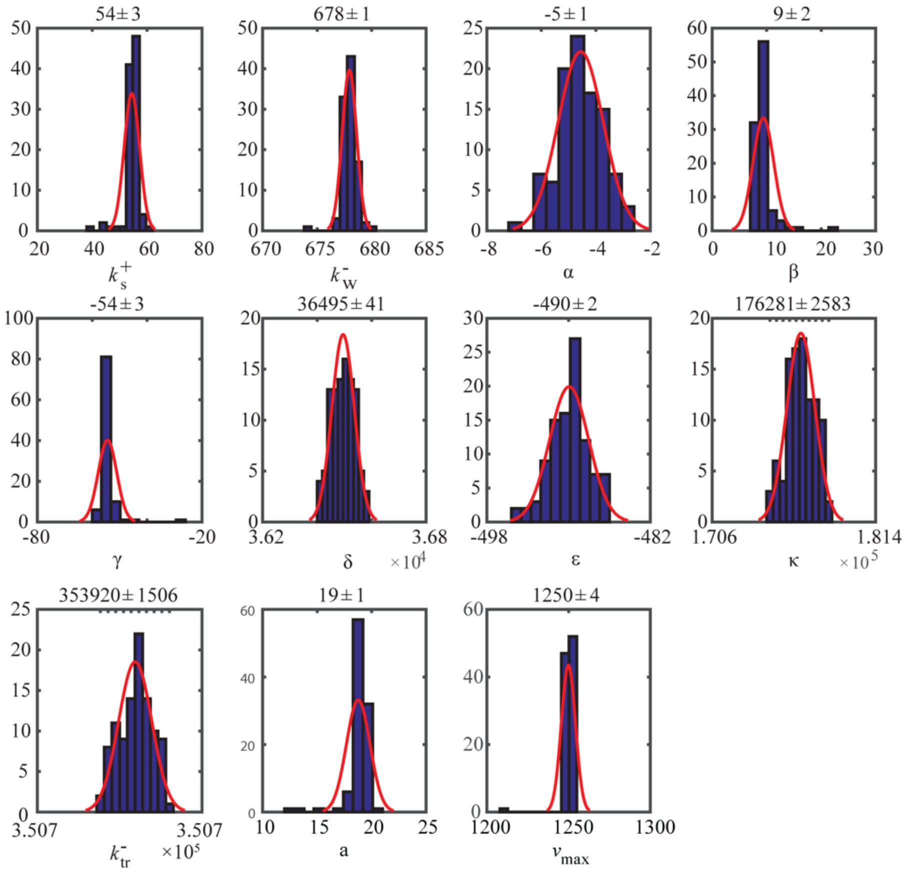

Supplementary Fig. 23: Model robustness analysis. Distribution of the fitted parameters calculated by a Monte Carlo bootstrap test from 100 simulated datasets. Red curves through the data are best fits to Gaussian distributions. The values on top of the histograms represent their mean ± width.

Supplementary references

1. Coluccio, L. M. *Myosin- A Superfamily of Molecular Motors*. *Myosins* (Springer Netherlands, 2007). doi:10.1007/978-1-4020-6519-4_4.
2. Henn, A., Cao, W., Hackney, D. D. & De La Cruz, E. M. The ATPase Cycle Mechanism of the DEAD-box rRNA Helicase, DbpA. *J. Mol. Biol.* **377**, 193–205 (2008).
3. KJ, Moore. & TM, Lohman. Kinetic mechanism of adenine nucleotide binding to and hydrolysis by the Escherichia coli Rep monomer. 1. Use of fluorescent nucleotide analogues. *Biochemistry* **33**, 14550–14564 (1994).
